# In situ ptychographic x-ray nanotomography of temperature-controlled crystallization processes

**DOI:** 10.1038/s41467-026-73738-1

**Published:** 2026-05-29

**Authors:** Zhao Jiang, Zirui Gao, Christian Appel, Maxime Durelle, Thomas Turner, Andreas Menzel, Alexander N. Kulak, Yi-Yeoun Kim, Manuel Guizar-Sicairos, Mirko Holler, Fiona C. Meldrum, Johannes Ihli

**Affiliations:** 1 School of Chemistry, University of Leeds, Leeds, UK; 2 Department of Chemistry, Durham University, Durham, UK; 3 National Synchrotron Light Source II, Brookhaven National Laboratory, Upton, New York, USA; 4 Photon Science Division, Paul Scherrer Institut, Villingen PSI, Switzerland; 5 Institute of Physics (IPHYS), École Polytechnique Fédérale de Lausanne, Lausanne, Switzerland; 6 Experiments Division, ALBA Synchrotron, Cerdanyola del Vallès - Barcelona, Spain

**Keywords:** Imaging techniques, Solid-state chemistry

## Abstract

Dynamic processes such as crystallization, sintering and phase separation play pivotal roles in defining the structure and performance of engineered and natural materials. Yet, these phenomena are often challenging to study because they are transient, spatially heterogeneous, and span multiple length and time scales. Visualizing them in three dimensions under realistic conditions therefore requires imaging techniques capable of probing representative sample volumes with nanoscale resolution and minute-scale temporal resolution, sustained over extended observation times and across a wide range of environmental conditions, capabilities that current in situ methods rarely combine. Here, we present an integrated platform for in situ time- and temperature-resolved ptychographic X-ray nanotomography that meets these demands, and demonstrate its capability by tracking the crystallization of amorphous calcium carbonate from room temperature to 500°C. Quantitative tomograms are acquired at five-minute intervals, producing a 4D dataset that reveals multiple simultaneous crystallization pathways, and rare and transient events. Among these is the formation and recrystallization of a metastable polymorph, calcium carbonate hemihydrate, which has previously only been observed in additive-stabilized systems. We also demonstrate how volume defect evolution and structural rearrangements within individual crystals contribute to the mechanisms underlying Ostwald ripening. This platform offers a general method for in situ visualization of material transformations, providing insights into the processes that govern material structure and functionality.

## Abstract (web summary)

Dynamic, temperature-resolved ptychographic X-ray nanotomography enables 3D tracking of material transformations such as crystallization, revealing multiple pathways, rare transient intermediates, and underlying mechanisms.

## Introduction

Imaging dynamic transformations of materials, as they occur, in three dimensions and under representative conditions, e.g., pressure, temperature or atmosphere, is essential to understand the mechanisms that govern function, failure, and long-term performance^1–5^. Many materials of technological and environmental importance, including catalysts^5,6^, batteries^1,7^, ceramics^8^, biominerals^9,10^, and geological systems^2,11,12^, are hierarchical composites whose behavior is shaped by nanoscale structure and local heterogeneities. Their evolution involves coupled structural and compositional changes, such as phase transitions, coarsening, fracture propagation, and defect formation, that unfold transiently across broad spatial and temporal scales. These transformations are often sparse, pathway or local environment-dependent, and can proceed through metastable intermediates that are invisible to conventional characterization methods. Capturing these transformations and their dependencies requires in situ imaging approaches that combine nanometer spatial resolution, minute-scale temporal resolution or better and the sensitivity to detect subtle changes within extended, system-representative volumes.

While many characterization techniques have been adapted for in situ or operando studies, none yet fully satisfy these combined demands. Atomic force microscopy, transmission electron microscopy, and Bragg coherent diffraction imaging offer exceptional spatial resolution, but are typically limited to planar samples, isolated crystals or sub-micrometer volumes^13–20^. Bulk techniques, including spectroscopy and scattering, provide statistically representative results but cannot resolve spatial dependencies and often fail to capture rare and or transient events^21–24^. Full-field micro-X-ray tomography has become a central tool for in situ and operando studies due to its ability to rapidly examine millimeter-sized samples at micrometer resolution and its straightforward integration with diverse sample environments^3,7,25,26^. By contrast, full-field in situ X-ray nanotomography, which enables rapid examination of micrometer-sized samples at tens to hundreds of nanometers spatial resolution, remains comparatively rare^26–32^. Achieving such resolutions typically relies on nanofocusing optics, which introduce trade-offs between field-of-view and spatial resolution, limit depth of field, and impose tight spatial constraints, complicating the integration of environmental control systems and iterative nano-sample-position/ correction instrumentation needed for extended measurements under varying conditions. Scanning nanoprobe X-ray tomography offers even higher spatial resolution, but long acquisition times, and similar stringent optical and stability requirements, have largely restricted studies to ex situ conditions and small volumes^33^. X-ray ptychography and its tomographic extension, ptychographic X-ray computed tomography (PXCT)^34–36^, are scanning, lensless, coherent diffraction imaging techniques that are maturing into general user methods at synchrotron facilities. PXCT enables the acquisition of quantitative electron-density tomograms of extended sample volumes with spatial resolution limited not by X-ray optics but by the recovered scattering signal and available coherent flux, and can, in principle, approach wavelength-limited resolution. This allows the use of micrometer-scale illumination probes while retaining nanometer spatial resolution and quantitative contrast across extended sample volumes, providing practical advantages such as an effectively extended depth of field, robustness against optical aberrations, high dose efficiency, and flexibility in sample geometry and experimental design. However, because ptychography yet remains a scanning technique, tomographic implementations have so far been largely restricted to ex situ studies due to yet long acquisition times and the lack of integrated environmental control and sample-positioning/correction instrumentation.

Here, we present an integrated platform for in situ dynamic ptychographic X-ray nanotomography that combines near-field X-ray ptychography^34–36^, angularly sparse tomogram acquisition, dynamic tomographic reconstruction^9,37^, and a custom environmental control and nano-positioning instrument^38^. This platform enables in situ ptychographic tomography measurements with nanometer spatial and minute-scale temporal resolution of extended sample volumes under defined temperature and gas environments, without the need for manual intervention or repeated sample re-centering over extended experimental durations. These capabilities are demonstrated by tracking the crystallization of amorphous calcium carbonate (ACC)^13,14,37,39,40^ over a 25-h heating cycle from room-temperature up to 500°. ACC offers a perfect example of a dynamic, multiscale process that is ubiquitous in natural and industrial systems^9,39,41^. ACC draws significant attention due to its importance in geology, CO₂ sequestration and biomineralization. However, due to the complexity of its crystallization mechanism^9,10,39,41^, where ACC can transform into calcite via classical and non-classical routes including progressive dehydration and the formation of intermediate crystalline phases^9,10,14,39,40,42–45^, many questions remain regarding the pathways by which this occurs. In common with all crystallization processes, multiple nucleation and growth events occur simultaneously within a given volume^10,41,46–48^, and the local environment of a nascent crystal—as defined by the number, size, location and structure of adjacent particles—will influence its crystallization pathway^10,41,46–48^. Samples undergoing crystallization are therefore expected to exhibit heterogeneous structural and compositional changes that cannot be fully captured by analytical techniques that average over the entire sample volume.

Using in situ ptychographic nanotomography to quantitatively track the crystallization of ACC, we reveal that the system is indeed highly heterogeneous and identify both dominant crystallization pathways and rare, transient phenomena. Hydrated ACC is seen to undergo stepwise dehydration before transforming into calcite, either directly or via metastable phases^13,37,42,43^. Importantly, we show that the transformations proceed via multiple pathways that are dependent on the local environment, as exemplified by the observation of rare events such as the ripening of defect-rich calcite single crystals and the formation of short-lived calcium carbonate hemihydrate, a phase that has previously only been observed in systems containing additives^49,50^. This work, therefore, establishes a general platform for imaging dynamic, multiscale transformations in reactive systems that narrows the information gap between electron microscopy and bulk techniques, while also providing new insight into the crystallization of ACC. This will create new opportunities for mechanistic studies of complex materials systems.

## Results

### In situ near-field ptychographic X-ray computed nanotomography

**Fig. 1:**
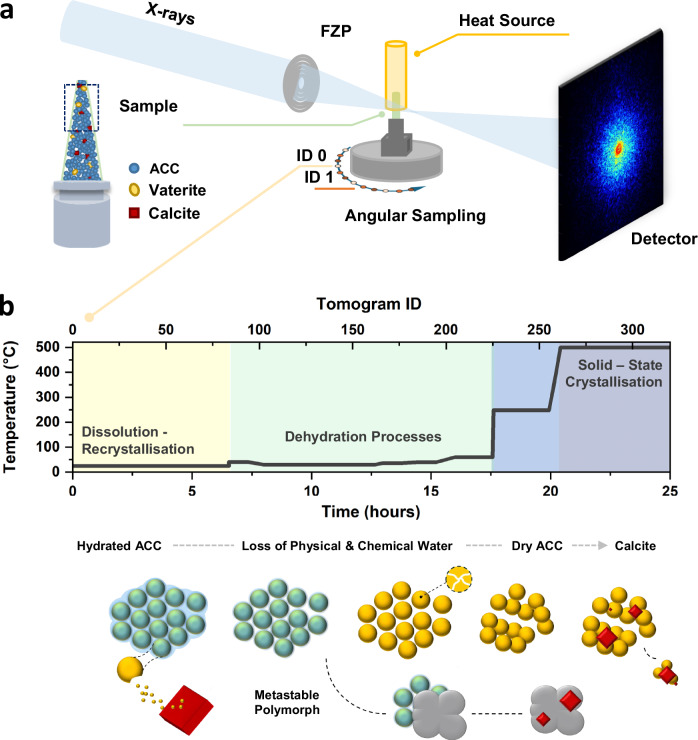
In situ dynamic near-field ptychographic X-ray computed nanotomography. **a** Schematic of the in situ setup. Tomographic projections were acquired via near-field X-ray ptychography. A Fresnel zone plate (FZP) was used for sample illumination. The sample was scanned in the x-y plane, acquiring a far-field diffraction pattern at each scanning position. Ptychographic reconstruction of these spatially overlapping patterns yielded the tomographic projections. Projections were acquired using a nested approach, in which sets of 10 equally spaced projections (ID) were iteratively collected with an increasing angular offset over 180°, for 25 h (dotted lines). A dynamic reconstruction algorithm was used for the tomographic reconstruction of all projections, resulting in 320 high-resolution electron density tomograms, one for every set of 10 projections. Heating was provided by a gas stream directed at and constrained to the sample and regulated via an environmental control system (orange tube). Examined was the crystallization of amorphous calcium carbonate (ACC) aggregates within a tapered capillary (blue box shows the field of view ~ 80 × 50 μm) as a function of time and temperature. **b** Time–temperature profile during data acquisition, showing the applied temperature ramp and number of acquired tomograms (Tomo ID). Shaded regions mark temperature ranges associated with distinct crystallization steps of ACC and are illustrated below. Source data are provided as a Source Data file.

In situ ptychographic nanotomography was performed under controlled conditions using a custom-built positioning instrument equipped with an environmental control system (Fig. 1a). This setup allows selective sample heating up to 850 °C and control over the sample surrounding gas atmosphere composition, while maintaining 360° rotational freedom and sub-10 nm sample positioning accuracy; critically, over extended durations and across the full temperature range^38^. The commonly spatial-resolution and field-of-view deteriorating sample drift is minimized by mechanically decoupling the heat source from the actively cooled, interferometer-corrected sample positioning stage.

A tapered capillary filled with aggregates of hydrated ACC was mounted on the instrument (Figs. S1–S3)^51^. The sample was heated stepwise from 25 °C, to 60 °C, 250 °C and 500 °C over 25 h, each temperature was maintained for > 2 h (Fig. 1b). This protocol allowed us to probe key stages of the crystallization of ACC, from crystallization mediated by surface-bound water at 25 °C, to physical changes accompanying the loss of physically- and chemically-bound water at 60 °C and 250 °C, to its solid-state crystallization to calcite at 500 °C^40,43^. 3,208 ptychographic projections were acquired over this 25-h period using a nested acquisition strategy. The projections consisted of angularly offset groups of 10 projections, each acquired evenly spaced over 180°. Tomographic reconstruction of all projections, using a dynamic reconstruction algorithm^52,53^ that leverages temporal continuity, yielded 320 high-resolution electron density tomograms, i.e., one per group. These tomograms have a voxel size of 82.84 nm and a sample-average spatial resolution of ~130 nm, as determined by Fourier shell correlation analysis using the half-bit threshold criterion (Figs. S4, S5). Under the two-pixel sampling expectation, this corresponds to an average spatial resolution of ~160 nm. Although one tomogram was obtained every 5 min, a conservative estimate places the sample-average temporal resolution at ~25 min (Fig. S6), representing a greater than 100-fold improvement over analytical reconstruction methods^54^. The representativeness of the in situ experiment (Fig. S7) and the validity of the dynamic reconstruction process were verified (Figs. S8–10). Experimental details are provided in the Methods Section.

### A spatially resolved view of dehydration and crystallization

**Fig. 2:**
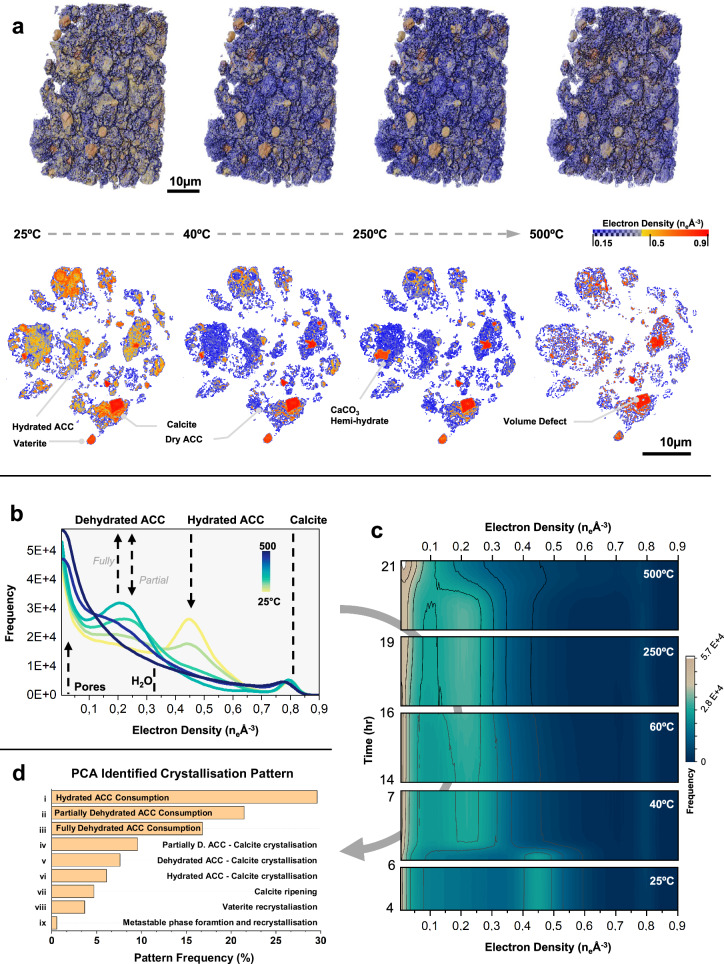
A spatially resolved view of amorphous calcium carbonate crystallization. **a** Volume renderings of and orthoslices through selected electron density tomograms of the same amorphous calcium carbonate (ACC) deposits at 20 °C, 60 °C, 250 °C, and 500 °C. The tomograms represent the pristine state, post-loss of physically and chemically bound water, and solid-state crystallization. Voxel size: 83 nm. Capillary walls are masked. Volume renderings and orthoslices share a common diverging color map corresponding to the local electron density, centered on the electron density of hydrated ACC; upon heating, the system evolves towards both higher and lower electron density components, and the color map highlights these deviations. The color map includes a semi-transparent range set to 30% (indicated by the checkered background in the legend) to improve visual accessibility of the volume renderings. **b** Selected 1D electron density histograms. Theoretical electron densities of selected sample components are indicated. **c** Corresponding 2D electron density histogram highlighting global changes in sample composition as a function of time and temperature. **d** Dominant crystallization pattern reoccurring throughout the sample volume. These patterns, derived from voxel-level PCA analysis, correspond to groups of voxels exhibiting similar electron-density changes with time and temperature (Fig. S13). Source data are provided as a Source Data file.

Figure 2a shows volume renderings and orthoslices of four tomograms selected from the 320 recorded. These show the pristine sample at 25 °C, the sample following the loss of physically- and chemically-bound water at 40 & 250 °C, and after the solid-state crystallization to calcite at 500 °C (see also Movie S1).

At 25 °C, the sample consists of micron-sized aggregates of hydrated ACC (~0.47 e^−^ Å^−3^), surrounded by smaller regions of partially dehydrated, nanoporous ACC (~0.24 e^−^ Å^−3^), and a few isolated, defect-rich calcite crystals (~0.79 e^−^ Å^−3^ - Note S1, Table S1). Some structural changes were observed on incubating the sample at this temperature, including the growth of pre-existing calcite crystals via the dissolution of adjacent ACC particles, and limited dehydration of some ACC particles, where the dehydration front progressed inwards from the aggregate surfaces. The presence of two distinct hydration states, along with the unusually broad density distribution of the hydrated ACC, reveals a non-uniform hydration landscape and intrinsic nanoscale heterogeneity even in the as-synthesized material. This observation contrasts with the historical treatment of ACC as a single phase in both bulk characterization (Figs. S1, S9) and modeling studies^43,55–57^. At 60 °C, the remaining physically bound water is rapidly lost, converting hydrated ACC to a partially dehydrated ACC (~0.24 e^−^ Å^−3^). Pre-existing regions of partially dehydrated ACC remain largely unchanged. At 250 °C, chemically bound water is removed and fully dehydrated ACC (~0.19 e^−^ Å^−3^) forms. Despite these substantial changes, the sample morphology remains virtually unchanged, and significant new calcite formation is not observed, demonstrating that dehydration alone does not induce crystallization^40,43,55,56,58^. An increase in the temperature to 500 °C then causes extensive structural and compositional changes. Numerous solid-state crystallization events occur, forming calcite nanocrystals within the dehydrated ACC, causing the ACC aggregates to coarsen as its constituent particles are consumed (Figs. S8, S9). These calcite crystals remain largely sub-micron in size or grow slowly during the annealing period, due to the local depletion of ACC by competing nucleation sites (Fig. S11, Note S2). Simultaneously, pre-existing micron-sized calcite crystals grow larger and develop a more faceted and compact morphology. This growth is driven by the consumption of newly formed adjacent calcite and the transformation of loosely packed ACC particles at the crystal’s surface. The dataset further allowed us to explore the local environment that supports the formation of new calcite crystals. Correlation between the initial electron density of a voxel and its likelihood of crystallization showed that regardless of whether the pathway is solution-mediated or solid-state, calcite crystallization preferentially initiates from or within regions of dehydrated ACC (Fig. S12). This preferential nucleation in dehydrated ACC regions likely reflects a locally reduced barrier. Although ACC lacks long-range order, the lower hydration level may correlate with subtle short-range structural organization or densification, that can facilitate solid-state transformation or act as preferential sites for heterogeneous nucleation during dissolution–recrystallization.

The evolution of the 320 electron density histograms (Fig. 2b, c) provides a first quantitative view of these transformations. The correspondence between the histogram observed electron density peaks and material phases is summarized in Table S1. Initially, three discrete peaks are observed in the histogram, corresponding to hydrated ACC (~0.47 e^−^ Å^−3^), partially dehydrated ACC (~0.24 e⁻ Å⁻³), and calcite (~0.79 e^−^ Å^−3^). Heating shifts the distribution: hydrated ACC converts to partially dehydrated ACC at 40 °C, which is then replaced by fully dehydrated ACC (~0.19 e^−^ Å^−3^) upon further heating. These defined changes in density agree with ~10 wt.% of structural water in hydrated ACC^40,43,55,59^. At 250 and 500 °C, the calcite peak sharpens, and the histogram baseline gradually rises, which is consistent with the formation of calcite crystals and ongoing Ostwald ripening. The latter refers to a process in which larger crystals grow at the expense of smaller ones to reduce a system’s surface energy (Note S2). Visually, as shown in Fig. 2a, this manifests as the gradual disappearance of smaller crystals and the concurrent growth of larger ones within an increasingly porous sample volume. Notably, no significant changes in sample mass were observed during the observation period at 500 °C (Fig. S7), indicating that the observed structural evolution can be fully attributed to crystallization processes, with no evidence for oxidative or thermal decomposition of the CaCO_3_. Importantly, the ACC-related changes occur via the emergence and recession of discrete peaks rather than continuous density shifts in the histogram, which confirms that ACC—although amorphous—transitions between rather distinct hydration states. This behavior supports prior modeling and pair-distribution function studies suggesting that, despite the heterogeneity observed here on the micrometer to tens-of-nanometer scale (Fig. 2a), ACC contains well-defined short-range order motifs and microporous environments^40,55,60^.

Finally, common transformation patterns and rare crystallization events were identified via voxel-level principal component analysis (PCA) and k-means clustering (S13). PCA decomposes the 4D tomogram into orthogonal components that capture the largest sources of variance, so that each component corresponds to a dominant, recurring pattern of electron-density change as a function of temperature and time. These analyses allow us to identify, isolate and quantify systematic changes that, in their sum, describe a 4D tomogram. The resulting patterns can then be assigned to specific transformation or crystallization events (Fig. 2d) by considering, for example, their initial, intermediate and final electron densities, as well as their spatial distribution within the sample. Although most patterns relate to the ACC-to-calcite transition, PCA also captured rare events, including the presence and recrystallization of vaterite (see Note S3, Fig. S14), the formation and transformation of a hydrated metastable polymorph and the defect-driven ripening of calcite crystals, each discussed in detail below.

### Identification and characterization of a rare metastable polymorph

A key strength of in situ ptychographic tomography is its ability to detect rare or transient events, which, although often obscured in bulk analyses, can critically influence transformation pathways and material properties. This capability is particularly valuable in systems governed by metastability, such as pharmaceutical formulations or functional ceramics^61,62^.

**Fig. 3:**
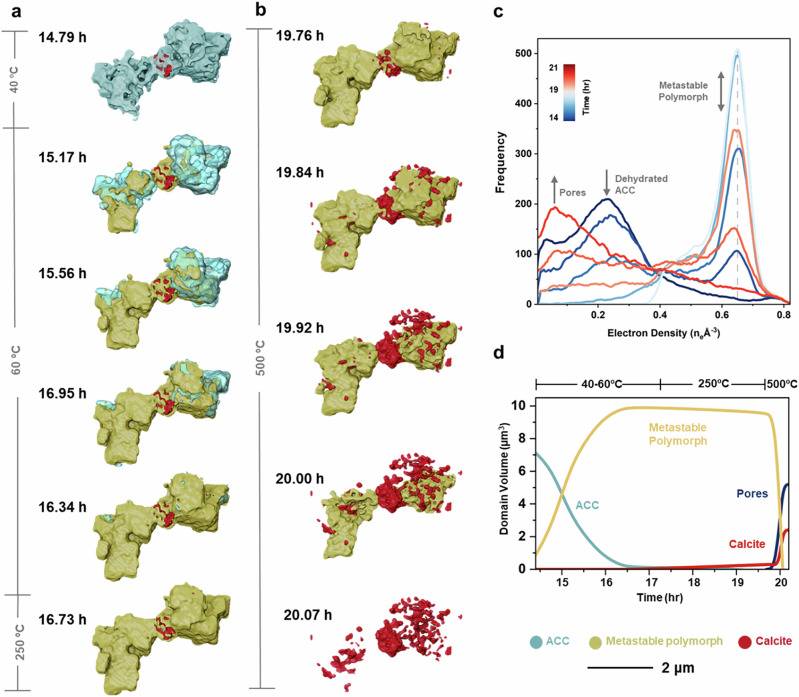
Formation and recrystallization of a rare metastable polymorph crystal. **a** Volume renderings showing the formation of a metastable CaCO₃ polymorph crystal (yellow) from amorphous calcium carbonate (ACC, teal) as a function of time and temperature. **b** Volume renderings showing the recrystallization of the crystal into calcite (red). Volume renderings are restricted to voxels at one point occupied by the metastable phase. **c** Selected electron density histograms detailing the formation and recrystallization of the metastable polymorph. **d** Plotted is the volume and the average electron density as a function of time and temperature. Source data are provided as a Source Data file.

Here, we resolve one such metastable phase, which comprises less than 0.5% of the total sample volume, and forms micrometer-sized faceted crystals. Figure 3a illustrates the formation of one of these metastable crystals, while Fig. 3b shows its subsequent recrystallization into calcite. On heating to 60 °C, a faceted crystal roughly 20 μm³ in volume forms rapidly by consuming the surrounding partially dehydrated ACC. The crystal possesses a uniform electron density of 0.66 e^−^ Å^−3^ throughout its formation. The density is consistent with that of calcium carbonate hemihydrate^49,50^ (Table S1). The crystal forms adjacent to and eventually enwraps a distinct high-density region, suggesting a heterogeneous nucleation origin, possibly on a calcite crystal (Movies S2, 3). The hemihydrate then remains structurally intact up to 500 °C, at which point it recrystallizes into calcite via multiple nucleation events (Fig. 3c).

The observation of calcium carbonate hemihydrate is surprising and fascinating. Being a rather recently discovered form of calcium carbonate^49,50^, it has previously only been observed in spatially confined systems or those containing phase-stabilizing additives such as magnesium ions^49,50,63^. Its emergence here therefore raises two possibilities, (i) that hemihydrate is a more common intermediate but is seldom detected due to its short lifetime or low abundance, or (ii) that the local conditions in our experiment, e.g. restricted mass transport, favor its formation. In either case, our observations highlight how nanoscale environments can selectively promote the formation of metastable polymorphs that are rarely accessible in bulk or ex situ studies.

### Thermal coarsening and defect evolution in single crystals

In situ ptychographic nanotomography at elevated temperatures further enabled the visualization of nanoscale defects within crystals by the detection of local electron-density deviations, and their role in the Ostwald ripening of crystalline materials. Such coarsening processes are widespread in materials science and often govern the evolution of microstructures^64,65^.

**Fig. 4:**
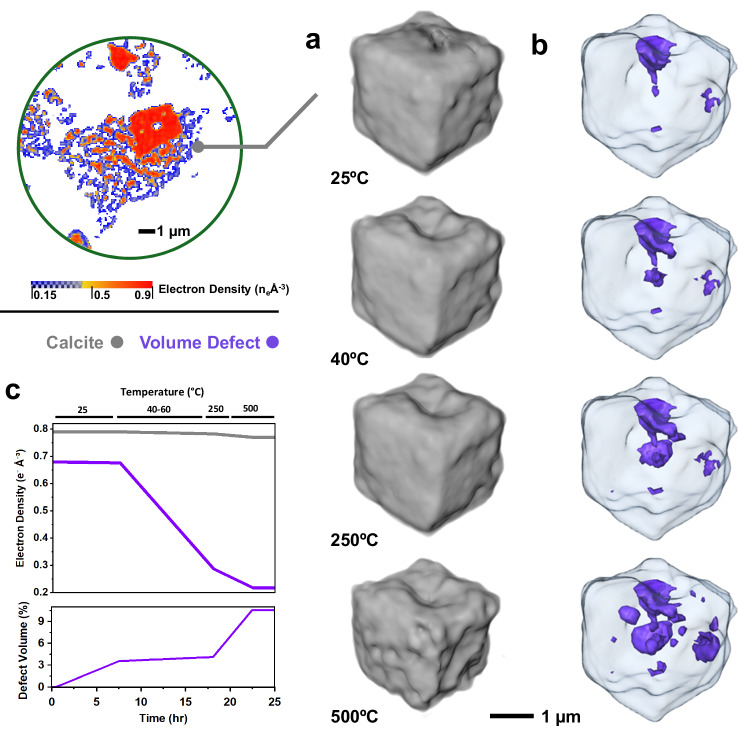
Defect evolution during the thermal ripening of a single crytal. **a** Volume renderings of a defect-rich calcite crystal at increasing temperatures. The region highlighted by the green circle shows part of an orthoslice through the final electron density tomogram acquired at 500 °C, indicating the crystal surroundings. Its location within the larger sample volume is shown in Fig. 2a. The volume renderings display the exterior of the isolated crystal in greyscale. A segmentation threshold of 0.75 n_e_ Å^−3^ was applied to isolate the crystal. **b** Corresponding renderings of the crystal’s internal volume defects. **c** Quantitative evolution of the defect volume (bottom) and mean electron density of defect and crystal (top). The central defect is already present at room temperature; increasing temperature leads to the gradual annihilation of the defect-rich material and structural reordering/faceting of the crystal. Source data are provided as a Source Data file.

Figure 4a, c show the evolution of a ~ 5 µm calcite crystal containing a central volume defect from room temperature to 500 °C. At room temperature, the crystal exhibits a low-density core (0.68 e^−^ Å^−3^ compared to the surrounding 0.79 e^−^ Å^−3^ calcite lattice). Although the defect is primarily confined to the crystal interior, faint extensions reach the crystal surface (Fig. S6). The crystal remains unchanged upon heating to 60 °C, but at 250 °C, the defect rapidly expands to ~580 nm in diameter, while its density decreases to 0.30 e^−^ Å^−3^ and secondary defects such as cracks form in the calcite crystal. These transformations accelerate at 500 °C. The central defect becomes larger, more-defined and continues to decrease in density, resulting in a hollow nanoporous crystal interior. In addition, smaller defects (~0.69 e^−^ Å^−3^) nucleate throughout the crystal, and faceted, geometric surfaces develop, consistent with Ostwald ripening (see also Movies S4). Notably, while isolated regions of the calcite crystal reach electron densities of 0.82 e^−^ Å^−3^ upon heating to 500 °C, indicative of a defect-free calcite, the average density remains stable at ~0.79 e^−^ Å^−3^ (Fig. 4d).

The location, size and morphology of the central defect suggest that the crystal initially nucleated on and then grew around an ACC particle. This is supported by the timing and scale of mass loss and crack formation at 250 °C, which is consistent with water being released from partially dehydrated and crystalised ACC^58^. The appearance of new, smaller volume defects at 500 °C throughout the crystal likely arises from the migration and coalescence of point defects, such as hydroxyl groups (OH^-^) substituting for carbonate ions in the lattice. The coalescence of defects and upon reaching a critical concentration, can lead to crystal lattice collapse, volume defect generation and the release of trapped and forming species such as CO₂ and H₂O^58,66^.

## Discussion

X-ray nanotomography has historically been constrained to static or ex situ treated samples. In situ or operando implementations remain rare, due to challenges in maintaining nanometer stability over long durations, or under in situ conditions and the risk of sample damage from the high X-ray doses required for nanoscale imaging. The most common approaches to X-ray nanotomography are scanning nanobeam (or nanoprobe) tomography and full-field nanotomography. Scanning X-ray nanoprobes offer high spatial resolution but are resolution-limited by X-ray focusing optics and are yet prohibitively slow. Acquisition times scale poorly with sample volume and resolution due to the need for raster scanning via a resolution-limiting probe^33,54^. Full-field nanotomography enables faster volumetric imaging by capturing entire projections simultaneously, but is equally limited by optical constraints, resulting in a trade-off between spatial resolution and field-of-view^26,28,29,67,68^.

The approach presented here, combining X-ray ptychography with angularly sparse data acquisition, dynamic tomographic reconstruction (lessening limitations in acquisition speed, dose, and optical constraints)^18,27,52,69,70^, and precision environmental control (alleviating stability concerns)^38^, reduces the barriers towards controlled in situ nanotomography experiments across broad environmental conditions and extended observation periods. By enabling in situ, time-resolved, quantitative, nanotomography of system-representative volumes and under controlled conditions, we can capture dominant and rare transformations as they unfold and build a comprehensive view of structure, dynamics and heterogeneity. This narrows the longstanding gap between electron microscopy and bulk investigations^11,13,16,18,21,25,27,49^. The approach has excellent generality, where it can be applied regardless of the composition and crystallinity of a material, and is highly sensitive, allowing us to study processes even in materials made of lighter elements. Possible applications range from investigations of lithium plating in solid-state batteries, where rare interfacial heterogeneities determine failure^7,71^, to grain and pore coarsening in alloys and ceramics, where microstructure shapes material properties^72^, and pharmaceutical development, capturing fleeting polymorphs and amorphous precursors that govern drug solubility and bioavailability^61,62^.

The capabilities of the approach were validated by tracking the crystallization of micron-sized ACC aggregates over a 25-h heating cycle. The dominant transformation pathway involved progressive dehydration at 60 °C and 250 °C, followed by extensive solid-state crystallization at 500 °C. Notably, transformations proceeded heterogeneously throughout the sample, governed by nanoscale variations in hydration state and structure^43,55,56,59,73^. We also captured the formation and recrystallization of calcium carbonate hemihydrate, which is a rare intermediate previously observed only in additive-stabilized systems^49,50,63^, and revealed how defects governed the ripening of calcite single crystals. The latter process involved vacancy migration, defect coalescence, void formation and the creation of surface facets, which are all hallmarks of Ostwald ripening^65^.

These first in situ experiments also highlight current limitations of the approach. Tomogram acquisition times and effective temporal resolution are on the order of several to tens of minutes for the here demonstrated sample volume and spatial resolution. Both spatial and temporal resolution were dominantly limited by the coherent flux of the third-generation synchrotron source and the sample-to-detector distance, necessitating point acquisition times of tens of milliseconds, and restricting scanning probe dimensions/ respectively, the use of near-field ptychography. The coherent flux of fourth-generation synchrotron sources^74^, and optimized beamlines is expected to improve temporal resolution. When combined with multi-beam illumination and multi-slice reconstruction strategies^75–77^, further relaxing lateral and angular sampling requirements, these advances are projected to enable sparse tomogram acquisition times at or below the one-minute scale for the demonstrated sample volume. These developments also impact radiation dose. Although ptychography with angularly sparse sampling is already dose-efficient, increased coherent flux and extended observation times raise concerns not only for canonically considered radiation-sensitive materials, but also for fine-structured materials, including integrated circuits^78^. Larger probes, relaxed sampling, and non-rigid reconstruction approaches capable of accommodating radiation-induced sample changes^52^ are therefore likely to be critical. Improvements in flux, data acquisition & reconstruction, alongside detector technology, are further expected to improve spatial resolution, enabling sub-10 nm resolution for these sample volumes.

Finally, adaptive or event-driven data acquisition routines are crucially missing from the present demonstration^79–81^. In such schemes, imaging parameters, including exposure time, angular sampling, field-of-view or environmental conditions such as temperature, are dynamically adjusted in response to environment-driven changes in the sample or experimental setup. Their absence, as illustrated here, can lead to a reduced effective field-of-view or unnecessary data acquisition and radiation dose during quiescent periods, as evident in Fig. 2c. Implementation of such routines will require near-real-time ptychographic image reconstruction and, ultimately, reconstruction and analysis of the evolving four-dimensional tomographic dataset. Depending on spatio-temporal resolution, volume and duration, such experiments can generate terabyte-scale datasets with estimated computational demands in the tera- to petaflop regime^81^. Apart from these HPC requirements, addressing this challenge will require automated analysis pipelines, incl. machine-learning-based methods for data reduction, and feature identification.

Beyond improvements in spatio-temporal resolution and dose efficiency, the approach offers opportunities for functional extension. The integration of spectroscopic contrast through combined sparse X-ray energy- and angle-resolved tomographic acquisition^53,69,82–86^ would enable simultaneous tracking of structural, compositional, and chemical evolution. Collectively, these developments position in situ ptychographic nanotomography as an evolving platform for the study of dynamic, multiscale transformations, including processes such as nucleation, spinodal decomposition, and phase-front propagation in reactive solids and liquids.

## Methods

### Materials

Analytical grade CaCl_2_·2H_2_O and Na_2_CO_3_ were purchased from Merck and used as received. CaCl_2_ and Na_2_CO_3_ stock solutions were prepared using Milli-Q water.

### Synthesis of amorphous calcium carbonate (ACC)

ACC was synthesized by mixing 2 mL of equimolar CaCl_2_ and Na_2_CO_3_ solution (200 mM). The resulting suspension of ACC particles was then filtered immediately through a 0.45 μm PVCT membrane and washed 2 times with 99.9% ethanol (~15 ml). The entire process took less than 5 min. The resulting powder consisting of ACC aggregates was transferred onto a filter paper for ~20 min to let the ethanol evaporate.

### Alignment and preparation of tapered capillaries

Tapered amorphous silica capillaries were fabricated with a capillary puller (Sutter Instrument P-2000). The tip diameter is approximately 4 μm, and the pulled capillary body is <100 μm in diameter. The capillaries were then secured in OMNY tomography pins, using UV-curable resin, with the capillary tip aligned with the OMNY pins center of rotation^51^.

### Loading and sealing of capillaries

The prepared ACC aggregates were gently broken up using a pestle and a mortar to ease the transfer into the tapered capillary. The powder was loaded into the backend of the capillary and moved to the capillary tip by placing the capillary on the sidewall of a (vibrating) ultrasonic bath. 200 µL of a 90/10 mixture of ethanol/water were then added to the backend of the capillary, and the backend was sealed with UV glue. The capillary tip was left open for the solvents and hydration water to escape during the in situ PXCT measurements. Ethanol/ water mixtures are known to stabilize ACC; the liquid was added to keep the hydration state of the ACC and prevent its crystallization during the sample transfer onto the instrument and the start of in situ measurements. Importantly, the ACC was synthesized on-site directly prior to the sample loading and PXCT measurement. The time between synthesis and the start of in situ PXCT experiments was <30 min. Shown in Fig. S2 is an optical micrograph of an ACC-filled tapered silica capillary.

### FTIR microscopy

The material loaded into the capillary was confirmed as ACC using Fourier-transform infrared (FT-IR) spectroscopy, where a spectrum was obtained from the loaded capillary directly prior to the tomography measurement (Fig. S1). Measurements were performed in transmission mode using a HYPERION 3000 microscope coupled to a Vertex 70 v spectrometer (Bruker), covering a spectral range of 4500–750 cm^−1^ using a local MIR source and a bolometer, located adjacent to the PXCT offering beamline. The beam size was ~50 μm, and spectra were averaged over 124 acquisitions while using a 4 cm^−1^ step size. Atmospheric contributions and instrumental background were removed.

### Ex-situ (Bulk) characterization

To support and validate the in situ tomography observations, ex-situ FTIR spectroscopy, SEM and PXRD measurements were carried out under conditions mimicking the in situ tomography experiment as far as possible. As-synthesized ACC was transferred onto glass slides, which were then placed into a furnace (Nabertherm Model No. LE 6/11/P300) programmed to replicate the temperature profile applied during the tomography measurements. Slides were removed from the furnace at specific time-points for analysis. FTIR spectra were acquired using a PerkinElmer Spectrum 100 AFT-IR Spectrometer. SEM was conducted by mounting a piece of the slide on an SEM stub using adhesive conducting pads, and the surface was coated with Ir (** ~** 1 nm thickness). Micrographs were acquired using a FEI Nova NanoSEM 450. Powder diffraction patterns were acquired using a PANalytical Empyrean diffractometer equipped with a Cu Kα source from 20° to 35° in intervals of 0.01, with a scan rate of 1° min^−1^ (Figs. S8, S9). We further acquired transmission electron micrographs of the synthesized sample and, after heating, to confirm the amorphous sample nature, ACC unit size and nanoscopic structural changes upon heating, Figs. S1, S16.

### In situ (near-field) ptychographic x-ray computed tomography (NF-PXCT)

PXCT, a combination of X-ray ptychography and computed tomography^36,87^, retrieves the complex-valued transmissivity of the examined sample, thereby providing access to quantitative tomograms of both phase and amplitude contrast with nanometer resolution^52,53^. When acquired far from sample-relevant absorption edges, the phase tomogram can be converted to a value-absolute electron density tomogram^88^.

#### Tomogram acquisition and reconstruction

NF-PXCT^34^ experiments were carried out at 6.2 keV at the cSAXS beamline of the Swiss Light Source, Paul Scherrer Institut, Switzerland. The photon energy was selected using a double-crystal Si(111) monochromator. The horizontal aperture of slits located 22 m upstream of the sample was set to ~20 μm in width, to create a horizontal virtual source point that coherently illuminated a Fresnel zone-plate, 200 μm in diameter, with an outermost zone width of 60 nm. The Fresnel zone-plate was designed with locally displaced zones to improve imaging quality^89^. The focus to sample distance was 6 mm, producing a focused probe of ~20 μm in diameter. Coherent diffraction patterns were acquired using an in-vacuum Eiger 1.5 M area detector, with a 75 µm pixel size, placed 5.229 m downstream of the sample inside an evacuated flight tube. Given the photon energy, sample to detector distance and detector pixel size, the far-field ptychography sampling condition would limit the maximum probe diameter to ~7 μm; the data were therefore reconstructed using a near-field propagator.

For sample positioning and environment regulation, we used the flexible tOMography Nano Imaging endstation (flOMNI) with modifications for environmental control. Details are provided in Holler et. al. (2022)^38,90^. A Fresnel zone plate scanner utilizing a combined motion of sample and illuminating FZP was used to achieve fast in-plane scanning speeds^91^. The field- of-view of each projection, or ptychographic scan, was 80 × 45 μm^2^ (height x width). The sample was scanned using a Fermat-spiral scanning pattern with an average step size of 5 µm, and the exposure time per scanning point was 0.05 s. For each ptychographic scan, a detector region of 1024 × 1024 pixels was used. The projection pixel size was 82.84 nm. Ptychographic reconstructions were performed using the PtychoShelves package with 500 iterations of the difference map algorithm followed by 600 iterations of maximum likelihood refinement^92^. Tomography projection acquisition followed a nested approach, where we acquired 10 equally spaced projections over 180° at a time, and then repeated the acquisition with an angular offset based on the golden ratio (Fig. 1a)^52,53^. The acquisition scheme was chosen to maximize the angular information diversity for the sparse or dynamic tomogram reconstruction.

Following ptychographic image reconstruction and spatial alignment of the projections^93^, tomographic reconstruction was performed using a dynamic sparse reconstruction technique, as first described in Gao et. al^52^. The technique allows dynamic tomography reconstruction with increased temporal resolution, which is comparable to the acquisition time of each sparsely sampled tomogram, i.e. each set of 10 projections, and without compromise in spatial resolution. It achieves this by exploiting temporal correlations, under the assumption that the sample evolves only minimally between successive time intervals (set of projections). In practice, a four-dimensional step-function model decomposes the sample dynamics into three constituent tomograms: the initial state, the final state, and a transition-time map. From these three components, one can then reconstruct a full, high-resolution tomogram for each individual time interval, without any a priori structural knowledge of the sample. In comparison to analytic tomographic reconstruction methods^54^, which require approximately 1250 projections to reconstruct a high-resolution tomogram of an 80 µm sample at 100 nm spatial resolution, the dynamic sparse reconstruction approach applied here achieves comparable image quality using only 10 projections per time point. This represents a > 100-fold enhancement in temporal resolution for in situ measurements, without compromising spatial resolution. A comprehensive benchmarking against the many existing iterative and compressed-sensing reconstruction methods is beyond the scope of this work; here we instead refer to the Crowther angular sampling criterion^54^ as a common analytical baseline for angular sampling, ensuring future comparativeness. Further considering that X-ray ptychography can achieve up to an order-of-magnitude higher dose efficiency than conventional scanning nanoprobes at fixed spatial resolution and image quality, and the ability to employ micrometer-scale scanning probes while retaining nm spatial resolution, this can reduce the number of scan positions required per resolution element by roughly one order of magnitude for the same field of view. When combined with the sparse angular sampling, this implies a substantial potential acquisition-speed advantage over conventional nanoprobe tomography for in situ measurements of the same volume and resolution. The exact factor will depend on source brightness, scan strategy and the performance of utilized instrumentation.

In total, we acquired 3208 projections, monitoring the dehydration, transformation, and crystallization of calcium carbonate from 25 to 500 °C (Fig. 1b). Tomographic reconstruction of the aligned projections was split into four time/temperature regimes, i.e., projections acquired at a set temperature/ across a temperature range were reconstructed together. This process was adopted to accommodate the large changes in the sample that occur on heating to 500 °C. These changes skew a combined reconstruction, reducing the spatial resolution and quantitativeness of the tomograms. The initial state of each transition period was set to match the final state of its precursor to ensure consistency across the whole process. 20000 iterations of iterative refinement based on tomographic forward- and back-projection were applied to the composite dynamic model.

To ensure positional stability during in situ heating, the environmental PXCT setup mechanically decouples the heat source from the actively cooled and interferometer-corrected sample positioning instrument^38,90^. This design minimizes thermally induced drift, providing sub–10 nm stability throughout data acquisition up to 850 °C. Any residual lateral drift of the sample’s center of rotation is continuously monitored and corrected in real time by the interferometer feedback system, with additional refinement during tomographic alignment. Vertical drift, primarily due to thermal expansion of the OMNY pin carrying the sample, remains unavoidable to a certain extent. This can be compensated for by shifting the vertical field-of-view in dependence on temperature. A routine to automatically shift the field-of-view was not yet implemented in the current study. The fixed field-of-view applied here during projection acquisition restricted tomographic reconstruction to a sample region/ height common to all projections across the full temperature series. As a result, the reconstructed tomograms encompassed only ~65% of the projection height. The integrated mass flow controllers of the environmental control system^38^ maintained a steady gas flow, ensuring a stable heating profile and consistent sample temperature. The flow rate was set to 1 L min^−1^, with temperature and humidity sensors placed approximately 2 cm from the OMNY pin tip. To facilitate continuous tomogram acquisition, a heating rate of 5 °C min^−1^ was applied for each incremental temperature increase.

To validate the dynamic reconstruction process and confirm that the observed sparse metastable phase was not an artifact, we additionally reconstructed the acquired projections using a filtered back-projection approach, processing them in batches of 200 adjacent projections. The metastable phase was consistently observed in both reconstruction methods (Fig. S10).

### Spatial resolution estimates

Analysis of the tomograms focused on the phase component of the acquired PXCT dataset, i.e., the electron density tomograms, due to their superior spatial resolution and signal-to-noise ratio at the chosen X-ray energy compared to the absorption component (https://henke.lbl.gov/). Spatial resolution estimates of projections and tomograms were obtained using Fourier ring correlation (FRC) and Fourier shell correlation (FSC), respectively^94^.

To evaluate the spatial resolution of the acquired projections, we acquired two projections under identical conditions, e.g., at the same rotation angle, calculated the correlation between these two projections in the Fourier domain and estimated the spatial resolution based on the intersection of the correlation function with the one-bit threshold (see Fig. S4). FRC suggests a sample average spatial resolution of 113.45 nm.

The spatial resolution of the dynamically reconstructed electron density tomograms was estimated by first splitting the measured projections into two subsets, allocating every other projection to a respective set. Secondly, these two subsets were independently reconstrued as previously and compared using FSC. The FSC was calculated for 4 timepoints or tomograms out of the 320 time frames or tomograms, i.e. one for each temperature range. The spatial resolution was estimated based on the first intersection of the correlation function with the ½ bit threshold criterion function (see Fig. S5). The tomogram spatial resolution averaged across all time points and materials according to FSC is ~135 nm. The sample volume considered for FSC was restricted to calcium carbonate particles, i.e. excluding the capillary and the surrounding air. The FSC likely underestimates the spatial resolution for selected components, as the tomograms do contain voxel invariant in electron density across adjacent tomograms; further developments are needed to accurately assess the spatial resolution of 4D datasets.

### Time resolution of dynamically reconstructed tomograms

The maximum theoretical time resolution of the dynamic sparse tomography technique is determined by the time it takes to acquire a sub-tomogram, i.e., a set of angularly sparsely sampled projections over 180˚. Here, the acquisition of sub-tomogram consisting of 10 projections took ~5 min. To obtain a first estimate of the achieved time resolution, the acquired projections were again split into two independent sets of projections and reconstructed, calculating the “transition time” for each voxel and time frame per set of projections. The difference between the reconstructed transition times of the two sets then allows us to obtain a first method precision or temporal resolution estimate (Fig. S6). Similar to the spatial resolution estimate for 4D datasets, further exploration of the best techniques to evaluate temporal resolution is needed.

### Electron density uncertainty

The measurement baseline voxel-level uncertainty in the reconstructed electron density was estimated by calculating the standard deviation (σ) of the electron density of air surrounding the sample across multiple time frames. This yields a sample-averaged uncertainty of approximately 0.008 e^−^ Å^−3^, reflecting the measurement and reconstruction noise under static conditions and in non-changing sample regions. In dynamically evolving regions, additional uncertainty can arise from temporal averaging over the sub-tomogram acquisition window from ongoing material transformation and relocation during acquisition. The magnitude of this contribution depends on the local transformation kinetics and experimental conditions and cannot be quantified by a single global value. Accordingly, the reported uncertainty from surrounding air should be interpreted as a baseline estimate, with higher effective uncertainty expected locally in rapidly changing voxels.

### Setup temperature limitation and temperature uncertainty

The developed and here utilized environmental control setup^38^, enables controlled heating of samples from room temperature up to a verified temperature of 850 °C. Although the system could in principle operate at even higher temperatures ( ~ 900 °C), the upper limit was set at 850 °C to ensure stable operation, representative experimental conditions, and to prevent potential damage to nearby beamline components. The instrument maintains sub-10 nm sample positioning stability through mechanical decoupling of the heat source from the actively cooled and interferometer-corrected sample stage. While ensuring stability, a vertical temperature gradient is unavoidable, leading to a temperature-dependent deviation between the sample and the experimental set-point. The deviation is less at lower temperatures, <5 °C under 200˚ °C. At 500 °C, the sample is estimated to be ~20 °C cooler than the set value, whereas at 850 °C and in view of heat transfer limitations, deviations of up to 50 °C can occur (influenced by the thermal conductivity of the sample). Operation beyond this temperature would likely introduce further uncertainty, potentially compromising measurement representativeness.

### Tomogram dose estimation

The X-ray dose imparted during tomogram acquisition was estimated to be in the order of ~ 10^9^ Gy. This is based on the average area flux density of each ptychographic scan and the mass density of the specimen (assumed to be the density of calcite)^95^.

### Tomogram analysis

Post-reconstruction data analysis and visualization were carried out using MATLAB (MathWorks), ImageJ (NIH), and Avizo (Thermo Fisher Scientific).

### Material identification

Materials (ACC, calcium carbonate polymorphs, water and silica) were identified through cross comparison of measured electron densities obtained via PXCT with theoretical electron densities of likely sample constituents (Table S1). Partial volume effects, i.e., the co-occupation of a voxel by multiple materials resulting in volume-weighted electron densities, were considered during interpretation.

### Capillary and pore masking

Data analysis was confined to voxels containing CaCO₃ by masking both the silica capillary and permanently unoccupied pore space. Following spatial registration and resampling of the four dynamic tomograms onto a common grid, the SiO₂ capillary was segmented using thresholding (0.61–0.75 ne Å^−3^) and refined via morphological operations to exclude misclassified voxels. Misclassified voxel here refers to isolated voxels with electron densities in the segmentation range that are located within the interior of the capillary and likely represent CaCO₃ rather than silica. The resulting binary mask was inverted and applied to all tomograms. To identify all calcium carbonate locations across the dataset, a “CaCO₃-location mask” was generated by summing all tomograms and applying a threshold of 0.15 ne Å^−3^, thereby retaining only voxels that registered electron density above this value at any point. This mask was then used to exclude all permanently unoccupied voxels from subsequent analysis and visualization (see Fig. S15 for a pre-masking histogram).

### Aggregate mass changes

To confirm that the environmental conditions during the in situ tomography experiments were representative of bulk behavior, we compared the mass loss profile observed in the tomograms to that obtained by thermogravimetric analysis (TGA). Specifically, we calculated the change in integrated electron density within the CaCO₃-occupied sample volume as a function of temperature. The observed loss in electron density was subsequently converted to an estimated mass loss and directly compared to thermogravimetric analysis (TGA) data. Electron density losses below 250 °C, were attributed to the dehydration of ACC, while any losses at 500 °C were attributed to the release of CO₂. Voxels initially occupied by calcite were excluded from the analysis. The calculation assumes negligible net mass transport into or out of the tomographic field of view. The resulting mass loss profile showed excellent agreement with bulk TGA measurements (Fig. S7), confirming the representativeness of the experimental conditions.

### Voxel-level analysis

To identify the dominant crystallization patterns within the examined sample volume, we performed a Principal Component Analysis (PCA) at the voxel level. As a first step, the 4D tomogram was reshaped into a two-dimensional matrix, where each row represented a voxel and each column corresponded to the voxel’s electron density at a specific time point. Next, voxels with an electron density below 0.1 n_e_ Å^−3^ or those showing abrupt changes in density greater than 0.3 n_e_ Å^−3^ across a single time frame were excluded. PCA was then used to decompose this matrix ( > 98% of the voxel) into orthogonal components capturing the most significant variance across time/temperature. This yielded two outputs: (i) principal component coefficients representing the spatial distribution of each pattern across the sample, and (ii) component scores, which describe the temporal evolution of these patterns. When recombined, these components approximate the original dataset while reducing dimensionality, allowing the key crystallization pattern to be identified. To group voxels according to shared crystallization dynamics, i.e., reoccurring pattern of electron density changes with time in a voxel, we applied *k*-means clustering on the PCA scores and evaluated the optimal number of clusters (*k*) by calculating the Within-Cluster Sum of Squares (WCSS) for values of *k* between 1 and 14 (Fig. S13). The optimal number of components was determined through visual inspection, guided by the physical interpretability of both their spatial distribution and temporal signatures. This step was necessary because statistical approaches such as the elbow method, which rely on minimizing the Within-Cluster Sum of Squares (WCSS) to determine the optimal number of components, tend to overlook minor but here meaningful patterns.

### Formation and recrystallization of the metastable polymorph

To estimate the rates of formation and recrystallization of the metastable calcium carbonate polymorph (Fig. 3), we first created a mask encompassing all voxels that were occupied by this polymorph at any point during the measurement series. Within this isolated sub-volume, we then calculated the relative volumes of metastable polymorph, calcite, amorphous calcium carbonate (ACC), and pores at each time point using threshold-based segmentation. Voxels with an electron density <0.15 n_e_ Å^−3^ were classified as pores. Voxels with densities between 0.15 and 0.41 n_e_ Å^−3^ were classified as dehydrated ACC. Voxels with densities between 0.41 and 0.75 n_e_ Å^−3^ correspond to the metastable phase, and voxels with densities ≥ 0.75 n_e_ Å^−3^ were classified as calcite. Threshold selection was informed by the electron-density histograms shown in Fig. 3, which capture the local phase evolution with time and temperature. Changes in these volume fractions over time were used to determine the rates of both formation and recrystallization of the metastable polymorph. In addition, we extracted the average electron density of the metastable phase as a function of temperature to monitor its structural evolution (Fig. 3).

### Volume-defect mediated calcite growth and vaterite crystallization

The growth and transformation dynamics of vaterite (Fig. S14) and defect-rich calcite (Fig. 4), were quantified using the same volumetric analysis approach described for the metastable phase. Here, voxels with an electron density <0.15 n_e_ Å^−3^ were classified as pores. Voxels with densities between 0.15 and 0.75 n_e_ Å^−3^ were classified as vaterite. Voxels with densities ≥ 0.75 n_e_ Å^−3^ were classified as calcite. Specifically, the rates of vaterite recrystallization, calcite growth, and volume-defect-associated dissolution were determined by tracking changes in segmented voxel volumes over time within defined sub-regions.

## Supplementary information

Supplementary Information

Description of Additional Supplementary Files

Supplementary Movie 1

Supplementary Movie 2

Supplementary Movie 3

Supplementary Movie 4

Supplementary Movie 5

Supplementary Movie 6

Transparent Peer Review file

## Source data

Source Data

## Data Availability

The raw data, reconstructed projections and reconstruction codes generated in this study have been deposited in the Zenodo repository (10.5281/zenodo.15692857) and the SciCat Data Catalog (https://www.psi.ch/en/awi/scicat) under the proposal identifier (PiD), 20.500.11935/db610991-dfee-4929-84cb-e7355ee5292d. Source data are provided with this paper. The data can further be obtained from the authors upon request. Source data are provided with this paper.

## Code availability

The tomogram reconstruction and analysis codes/scripts used for this study can be further accessed at the following address https://www.psi.ch/en/sls/csaxs/software, or obtained from the authors upon request.

## References

[1] Liu, D. et al. Review of Recent Development of In Situ/Operando Characterization Techniques for Lithium Battery Research. *Adv. Mater.* **31**, 1806620 (2019). 10.1002/adma.201806620 31099081

[2] Aranda, M. A. G. 4D synchrotron X-ray nanoimaging for early age cement curing: where are we and where should we go? *Acc Mater. Res.* **6**, 814–827 (2025). 40741229 10.1021/accountsmr.5c00018 PMC12305653

[3] García-Moreno, F. et al. Tomoscopy: time-resolved tomography for dynamic processes in materials. *Adv. Mater.* **33**, 2104659 (2021). 34558111 10.1002/adma.202104659 PMC11468671

[4] Zhu, Y., Zhao, H., He, Y. & Wang, R. In-situ transmission electron microscopy for probing the dynamic processes in materials. *J. Phys. D: Appl. Phys.* **54**, 443002 (2021).

[5] Meirer, F. & Weckhuysen, B. M. Spatial and temporal exploration of heterogeneous catalysts with synchrotron radiation. *Nat. Rev. Mater.* **3**, 324–340 (2018).

[6] Petersen, H. & Weidenthaler, C. A review of recent developments for the in situ/operando characterization of nanoporous materials. *Inorg. Chem. Front.* **9**, 4244–4271 (2022).

[7] Ning, Z. et al. Visualizing plating-induced cracking in lithium-anode solid-electrolyte cells. *Nat. Mater.* **20**, 1121–1129 (2021). 33888903 10.1038/s41563-021-00967-8

[8] Palmero, P. Structural ceramic nanocomposites: a review of properties and powders’ synthesis methods. *Nanomaterials* **5**, 656–696 (2015). 28347029 10.3390/nano5020656 PMC5312897

[9] Meldrum, F. C. & Cölfen, H. The many lives of calcium carbonate. *Nat. Chem.* **15**, 1196–1196 (2023). 37488377 10.1038/s41557-023-01284-0

[10] Meldrum, F. C. & Cölfen, H. Controlling mineral morphologies and structures in biological and synthetic systems. *Chem. Rev.* **108**, 4332–4432 (2008). 19006397 10.1021/cr8002856

[11] Shirani, S. et al. 4D nanoimaging of early age cement hydration. *Nat. Commun.* **14**, 2652 (2023). 37156776 10.1038/s41467-023-38380-1 PMC10167225

[12] Regnet, J. B., David, C., Robion, P. & Menéndez, B. Microstructures and physical properties in carbonate rocks: a comprehensive review. *Mar. Pet. Geol.* **103**, 366–376 (2019).

[13] Nielsen, M. H., Aloni, S. & De Yoreo, J. J. In situ TEM imaging of CaCO3 nucleation reveals the coexistence of direct and indirect pathways. *Science* **345**, 1158–1162 (2014). 25190792 10.1126/science.1254051

[14] Smeets, P. J. M., Cho, K. R., Kempen, R. G. E., Sommerdijk, N. A. J. M. & De Yoreo, J. J. Calcium carbonate nucleation driven by ion binding in a biomimetic matrix revealed by in situ electron microscopy. *Nat. Mater.* **14**, 394–399 (2015). 25622001 10.1038/nmat4193

[15] Zhu, C. et al. In-situ liquid cell transmission electron microscopy investigation on oriented attachment of gold nanoparticles. *Nat. Commun.* **9**, 421 (2018). 29379109 10.1038/s41467-018-02925-6 PMC5788991

[16] Orme, C. A. et al. Formation of chiral morphologies through selective binding of amino acids to calcite surface steps. *Nature* **411**, 775–779 (2001). 11459051 10.1038/35081034

[17] Ihli, J. et al. Visualization of the effect of additives on the nanostructures of individual bio-inspired calcite crystals. *Chem. Sci.* **10**, 1176–1185 (2019). 30774916 10.1039/c8sc03733g PMC6349071

[18] Clark, J. N. et al. Three-dimensional imaging of dislocation propagation during crystal growth and dissolution. *Nat. Mater.* **14**, 780–784 (2015). 26030304 10.1038/nmat4320 PMC4623157

[19] Hata, S., Ihara, S., Saito, H. & Murayama, M. In-situ heating-and-electron tomography for materials research: from 3D (in-situ 2D) to 4D (in-situ 3D). *Microscopy* **73**, 133–144 (2024). 38462986 10.1093/jmicro/dfae008 PMC11000667

[20] Egerton, R. F., Li, P. & Malac, M. Radiation damage in the TEM and SEM. *Micron* **35**, 399–409 (2004). 15120123 10.1016/j.micron.2004.02.003

[21] Rodriguez-Blanco, J. D., Shaw, S. & Benning, L. G. The kinetics and mechanisms of amorphous calcium carbonate (ACC) crystallization to calcite, viavaterite. *Nanoscale* **3**, 265–271 (2011). 21069231 10.1039/c0nr00589d

[22] Stawski, T. M. et al. On demand” triggered crystallization of CaCO3 from solute precursor species stabilized by the water-in-oil microemulsion. *Phys. Chem. Chem. Phys.* **20**, 13825–13835 (2018). 29745416 10.1039/c8cp00540k

[23] Norby, P. In-situ XRD as a tool to understanding zeolite crystallization. *Curr. Opin. Colloid Interface Sci.* **11**, 118–125 (2006).

[24] Févotte, G. In situ raman spectroscopy for in-line control of pharmaceutical crystallization and solids elaboration processes: a review. *Chem. Eng. Res. Des.* **85**, 906–920 (2007).

[25] Derluyn, H. et al. Crystallization of hydrated and anhydrous salts in porous limestone resolved by synchrotron X-ray microtomography. *Nucl. Instrum. Methods Phys. Res. Sect. B: Beam Interact. Mater. At.* **324**, 102–112 (2014).

[26] Takeuchi, A. & Suzuki, Y. Recent progress in synchrotron radiation 3D–4D nano-imaging based on X-ray full-field microscopy. *Microscopy* **69**, 259–279 (2020). 32373929 10.1093/jmicro/dfaa022

[27] Lee, S. et al. Multiscale in-situ characterization of static recrystallization using dark-field X-ray microscopy and high-resolution X-ray diffraction. *Sci. Rep.* **14**, 6241 (2024). 38486085 10.1038/s41598-024-56546-9 PMC10940669

[28] Wang, J., Chen-Wiegart, Y. -CK. & Wang, J. In situ three-dimensional synchrotron X-ray nanotomography of the (De)lithiation processes in tin anodes. *Angew. Chem. Int. Ed.* **53**, 4460–4464 (2014). 10.1002/anie.201310402 24648150

[29] Vanpeene, V. et al. Comparative study of the quantitative analysis of battery materials with X-ray nano-tomography: from ex situ toward operando measurements. *ACS Nano* **19**, 9994–10012 (2025). 40040237 10.1021/acsnano.4c16419

[30] Flenner, S. et al. Hard X-ray full-field nanoimaging using a direct photon-counting detector. *J. Synchrotron Radiat.* **30**, 390–399 (2023). 36891852 10.1107/S1600577522012103 PMC10000802

[31] Longo, E. et al. X-ray Zernike phase contrast tomography: 3D ROI visualization of mm-sized mice organ tissues down to sub-cellular components. *Biomed. Opt. Express* **11**, 5506–5517 (2020). 33149967 10.1364/BOE.396695 PMC7587279

[32] Venkatesh, A. M., Bouvard, D., Lhuissier, P., Villanova, J. & Rajon, C. In-situ 3D X-ray investigation of ceramic powder sintering at the particle length-scale. *Ceram. Int.* **50**, 4715–4728 (2024).

[33] Mino, L. et al. Materials characterization by synchrotron X-ray microprobes and nanoprobes. *Rev. Mod. Phys.* **90**, 025007 (2018).

[34] Stockmar, M. et al. X-ray nanotomography using near-field ptychography. *Opt. Express* **23**, 12720–12731 (2015). 26074526 10.1364/OE.23.012720

[35] Stockmar, M. et al. Near-field ptychography: phase retrieval for inline holography using a structured illumination. *Sci. Rep.* **3**, 1927 (2013). 23722622 10.1038/srep01927 PMC3668322

[36] Miao, J., Ishikawa, T., Robinson, I. K. & Murnane, M. M. Beyond crystallography: diffractive imaging using coherent x-ray light sources. *Science* **348**, 530–535 (2015). 25931551 10.1126/science.aaa1394

[37] Bots, P., Benning, L. G., Rodriguez-Blanco, J.-D., Roncal-Herrero, T. & Shaw, S. Mechanistic insights into the crystallization of amorphous calcium carbonate (ACC). *Cryst. Growth Des.* **12**, 3806–3814 (2012).

[38] Holler, M. et al. Environmental control for X-ray nanotomography. *J. Synchrotron Radiat.* **29**, 1223–1231 (2022). 36073881 10.1107/S1600577522006968 PMC9455200

[39] De Yoreo, J. J. et al. Crystallization by particle attachment in synthetic, biogenic, and geologic environments. *Science* **349**, aaa6760 (2015). 26228157 10.1126/science.aaa6760

[40] Ihli, J. et al. Dehydration and crystallization of amorphous calcium carbonate in solution and in air. *Nat. Commun.* **5**, 3169 (2014). 24469266 10.1038/ncomms4169 PMC4085778

[41] De Yoreo, J. J. & Vekilov, P. G. Principles of crystal nucleation and growth. *Rev. Mineral. Geochem.* **54**, 57–93 (2003).

[42] Ihli, J., Kim, Y.-Y., Noel, E. H. & Meldrum, F. C. The effect of additives on amorphous calcium carbonate (ACC): janus behavior in solution and the solid state. *Adv. Funct. Mater.* **23**, 1575–1585 (2013).

[43] Radha, A. V., Forbes, T. Z., Killian, C. E., Gilbert, P. U. P. A. & Navrotsky, A. Transformation and crystallization energetics of synthetic and biogenic amorphous calcium carbonate. *Proc. Natl. Acad. Sci.* **107**, 16438–16443 (2010). 20810918 10.1073/pnas.1009959107 PMC2944757

[44] Gong, Y. U. T. et al. Phase transitions in biogenic amorphous calcium carbonate. *Proc. Natl. Acad. Sci.* **109**, 6088–6093 (2012). 22492931 10.1073/pnas.1118085109 PMC3341025

[45] Mass, T. et al. Amorphous calcium carbonate particles form coral skeletons. *Proc. Natl. Acad. Sci.* **114**, E7670–E7678 (2017). 28847944 10.1073/pnas.1707890114 PMC5604026

[46] Li, M., Chen, Y., Tanaka, H. & Tan, P. Revealing roles of competing local structural orderings in crystallization of polymorphic systems. *Sci. Adv.* **6**, eaaw8938 (2020). 32656336 10.1126/sciadv.aaw8938 PMC7329355

[47] Langer, J. S. Instabilities and pattern formation in crystal growth. *Rev. Mod. Phys.* **52**, 1–28 (1980).

[48] Meldrum, F. C. & O’Shaughnessy, C. Crystallization in confinement. *Adv. Mater.* **32**, 2001068 (2020). 10.1002/adma.202001068 32583495

[49] Zou, Z. et al. A hydrated crystalline calcium carbonate phase: calcium carbonate hemihydrate. *Science* **363**, 396–400 (2019). 30679371 10.1126/science.aav0210

[50] Schmidt, C. A. et al. Myriad Mapping of nanoscale minerals reveals calcium carbonate hemihydrate in forming nacre and coral biominerals. *Nat. Commun.* **15**, 1812 (2024). 38418834 10.1038/s41467-024-46117-x PMC10901822

[51] Holler, M. et al. OMNY PIN—A versatile sample holder for tomographic measurements at room and cryogenic temperatures. *Review of Scientific Instruments* **88**, 10.1063/1.4996092 (2017). 10.1063/1.4996092 29195351

[52] Gao, Z. et al. Dynamic sparse x-ray nanotomography reveals ionomer hydration mechanism in polymer electrolyte fuel-cell catalyst. *Sci. Adv.* **10**, eadp3346 (2024). 39383223 10.1126/sciadv.adp3346 PMC11463282

[53] Gao, Z. et al. Sparse ab initio x-ray transmission spectrotomography for nanoscopic compositional analysis of functional materials. *Sci. Adv.* **7**, eabf6971 (2021). 34108209 10.1126/sciadv.abf6971 PMC8189584

[54] Crowther, Richard Anthony, D. J. DeRosier, and Aaron Klug. The reconstruction of a three-dimensional structure from projections and its application to electron microscopy. *Proc. R. Soc. London A* **317**, 319–340 (1970).

[55] Nicholas, T. C. et al. Geometrically frustrated interactions drive structural complexity in amorphous calcium carbonate. *Nat. Chem.* **16**, 36–41 (2024). 37749235 10.1038/s41557-023-01339-2 PMC10774122

[56] Goodwin, A. L. et al. Nanoporous structure and medium-range order in synthetic amorphous calcium carbonate. *Chem. Mater.* **22**, 3197–3205 (2010).

[57] Michel, F. M. et al. Structural characteristics of synthetic amorphous calcium carbonate. *Chem. Mater.* **20**, 4720–4728 (2008).

[58] Schmidt, M. P., Ilott, A. J., Phillips, B. L. & Reeder, R. J. Structural changes upon dehydration of amorphous calcium carbonate. *Cryst. Growth Des.* **14**, 938–951 (2014).

[59] Gindele, M. B. et al. Colloidal pathways of amorphous calcium carbonate formation lead to distinct water environments and conductivity. *Nat. Commun.* **15**, 80 (2024). 38167336 10.1038/s41467-023-44381-x PMC10761707

[60] Clark, S. M. et al. The nano- and meso-scale structure of amorphous calcium carbonate. *Sci. Rep.* **12**, 6870 (2022). 35477728 10.1038/s41598-022-10627-9 PMC9046151

[61] H.G., B. *Polymorphism in Pharmaceutical Solids* 2edn, (CRC Press, 2009).

[62] Censi, R. & Di Martino, P. Polymorph impact on the bioavailability and stability of poorly soluble drugs. *Molecules* **20**, 18759–18776 (2015). 26501244 10.3390/molecules201018759 PMC6331817

[63] Suyama, M., Kitajima, T. & Fukushi, K. Solubility of calcium carbonate hemihydrate (CCHH): where does CCHH occur? *Geochem. Perspect. Lett.* **31**, 27–31 (2024).

[64] Fang, Z. Z., Wang, H. & Kumar, V. Coarsening, densification, and grain growth during sintering of nano-sized powders—A perspective. *Int. J. Refractory Met. Hard Mater.* **62**, 110–117 (2017).

[65] Baldan, A. Review progress in Ostwald ripening theories and their applications to nickel-base superalloys. Part I: Ostwald ripening theories. *J. Mater. Sci.* **37**, 2171–2202 (2002).

[66] Kim, Y.-Y. et al. An artificial biomineral formed by the incorporation of copolymer micelles in calcite crystals. *Nat. Mater.* **10**, 890–896 (2011). 21892179 10.1038/nmat3103

[67] Liu, X. et al. Formation of three-dimensional bicontinuous structures via molten salt dealloying studied in real-time by in situ synchrotron X-ray nano-tomography. *Nat. Commun.* **12**, 3441 (2021). 34108466 10.1038/s41467-021-23598-8 PMC8190292

[68] Withers, P. J. et al. X-ray computed tomography. *Nat. Rev. Methods Prim.* **1**, 18 (2021).

[69] Wirtensohn, S. et al. Nanoscale dark-field imaging in full-field transmission X-ray microscopy. *Optica* **11**, 852–859 (2024).

[70] Hua, W. et al. Probing particle-carbon/binder degradation behavior in fatigued layered cathode materials through machine learning aided diffraction tomography. *Angew. Chem. Int. Ed.* **63**, e202403189 (2024). 10.1002/anie.202403189 38701048

[71] Sadd, M., Xiong, S., Bowen, J. R., Marone, F. & Matic, A. Investigating microstructure evolution of lithium metal during plating and stripping via operando X-ray tomographic microscopy. *Nat. Commun.* **14**, 854 (2023). 36792892 10.1038/s41467-023-36568-z PMC9931753

[72] Praveen, S. & Kim, H. S. High-entropy alloys: potential candidates for high-temperature applications – an overview. *Adv. Eng. Mater.* **20**, 1700645 (2018).

[73] Schmidt, E. M. et al. Quantitative three-dimensional local order analysis of nanomaterials through electron diffraction. *Nat. Commun.* **14**, 6512 (2023). 37845256 10.1038/s41467-023-41934-y PMC10579245

[74] Streun, A. et al. SLS-2 - the upgrade of the swiss light source. *J. Synchrotron Radiat.* **25**, 631–641 (2018). 29714174 10.1107/S1600577518002722 PMC5929351

[75] Sidorenko, P. & Cohen, O. Single-shot ptychography. *Optica* **3**, 9–14 (2016).

[76] Nikitin, V. Carlsson, M. Mokso, R. Cloetens, P. & Gursoy, D. Single-distance nano-holotomography with coded apertures. *arXiv*10.48550/arXiv.2409.05163 (2024). 10.1364/OL.541765 39815565

[77] Åstrand, M. et al. Multi-beam multi-slice X-ray ptychography. *Sci. Rep.* **15**, 9273 (2025). 40102622 10.1038/s41598-025-93757-0 PMC11920106

[78] Aidukas, T. et al. High-performance 4-nm-resolution X-ray tomography using burst ptychography. *Nature* **632**, 81–88 (2024). 39085541 10.1038/s41586-024-07615-6

[79] Peters, J. J. P. et al. Event-responsive scanning transmission electron microscopy. *Science* **385**, 549–553 (2024). 39088619 10.1126/science.ado8579

[80] Szymanski, N. J. et al. Adaptively driven X-ray diffraction guided by machine learning for autonomous phase identification. *npj Comput. Mater.* **9**, 31 (2023).

[81] Babu, A. V. et al. Deep learning at the edge enables real-time streaming ptychographic imaging. *Nat. Commun.* **14**, 7059 (2023). 37923741 10.1038/s41467-023-41496-z PMC10624836

[82] Quinn, P. D. et al. Optimal sparse energy sampling for X-ray spectro-microscopy: reducing the X-ray dose and experiment time using model order reduction. *Chem. Biomed. Imaging* **2**, 283–292 (2024). 39473770 10.1021/cbmi.3c00116 PMC11503965

[83] Michelson, A. et al. Three-dimensional visualization of nanoparticle lattices and multimaterial frameworks. *Science* **376**, 203–207 (2022). 35389786 10.1126/science.abk0463

[84] Shukla, A. et al. Grain boundary strain localization in a CdTe solar cell revealed by scanning 3D X-ray diffraction microscopy. *J. Mater. Chem. A* **12**, 16793–16802 (2024).

[85] Shao, H. C., Mengke, T., Pan, T. & Zhang, Y. Dynamic CBCT imaging using prior model-free spatiotemporal implicit neural representation (PMF-STINR). *arXiv*10.1088/1361-6560/ad46dc (2023). 10.1088/1361-6560/ad46dc PMC11133878 38697195

[86] Apseros, A. et al. X-ray linear dichroic tomography of crystallographic and topological defects. *Nature* **636**, 354–360 (2024). 39663493 10.1038/s41586-024-08233-y PMC11634779

[87] Dierolf, M. et al. Ptychographic X-ray computed tomography at the nanoscale. *Nature* **467**, 436–439 (2010). 20864997 10.1038/nature09419

[88] Diaz, A. et al. Quantitative x-ray phase nanotomography. *Phys. Rev. B* **85**, 020104 (2012).

[89] Odstrčil, M., Lebugle, M., Guizar-Sicairos, M., David, C. & Holler, M. Towards optimized illumination for high-resolution ptychography. *Opt. Express* **27**, 14981–14997 (2019). 31163938 10.1364/OE.27.014981

[90] Holler, M. et al. OMNY—A tOMography Nano crYo stage. *Review of Scientific Instruments* **89**, 10.1063/1.5020247 (2018). 10.1063/1.5020247 29716370

[91] Odstrcil, M., Lebugle, M., Lachat, T., Raabe, J. & Holler, M. Fast positioning for X-ray scanning microscopy by a combined motion of sample and beam-defining optics. *J. Synchrotron Radiat.* **26**, 504–509 (2019). 30855261 10.1107/S160057751801785X PMC6412177

[92] Wakonig, K. et al. PtychoShelves, a versatile high-level framework for high-performance analysis of ptychographic data. This article will form part of a virtual special issue of the journal on ptychography software and technical developments. *J. Appl. Crystallogr.* **53**, 574–586 (2020). 32280327 10.1107/S1600576720001776 PMC7133065

[93] Odstrčil, M., Holler, M., Raabe, J. & Guizar-Sicairos, M. Alignment methods for nanotomography with deep subpixel accuracy. *Opt. express* **27**, 36637–36652 (2019). 31873438 10.1364/OE.27.036637

[94] van Heel, M. & Schatz, M. Fourier shell correlation threshold criteria. *J. Struct. Biol.* **151**, 250–262 (2005). 16125414 10.1016/j.jsb.2005.05.009

[95] Howells, M. R. et al. An assessment of the resolution limitation due to radiation-damage in X-ray diffraction microscopy. *J. Electron Spectrosc. Relat. Phenom.* **170**, 4–12 (2009). 10.1016/j.elspec.2008.10.008 PMC2867487 20463854

