# Supplementary material for: In situ ptychographic x-ray nanotomography of temperature-controlled crystallization processes
Source: Nat Commun. 2026 May 29;17:6994. doi: 10.1038/s41467-026-73738-1 (PMC13392228; doi:10.1038/s41467-026-73738-1)
Supplement: Supplementary file 1 — Supplementary Information [file 41467_2026_73738_MOESM1_ESM.pdf]

Supplementary Information

In Situ Ptychographic X-ray Nanotomography of Temperature-Controlled Crystallization Processes

The Supplementary Information contains:

Supplementary Note 1 to 3

Supplementary Figure 1 to 16

Supplemental Table 1

Supplementary References

Supplementary Note 1: Reduced Electron Density of Calcite Crystals

We observe an average electron density of 0.79 e^{-} $Å^{-3}$ for our calcite crystals, which is ~ 3.7% lower than the 0.82 e^{-} $Å^{-3}$ expected for defect-free calcite. The well-defined peak at 0.79 e^{-} $Å^{-3}$ in the electron-density histogram is inconsistent with an explanation rooted in crystal-internal nanodomains of water or residual ACC, which would yield broader and likely more asymmetric density distributions on a population basis.[1,2] Instead, the most plausible explanation is a systematic presence of point defects within the calcite lattice, either in the form of randomly distributed calcium (Ca^{2+}) or carbonate (CO_3^{2-}) vacancies, or minor substitution of carbonate by hydroxyl (OH^{-}) groups. A 3.7% density reduction corresponds to a defect concentration on the order of 2–7 at.% if entirely due to vacancies or substitution. While both explanations are plausible, we currently favour a vacancy-based explanation. Although hydroxyl groups readily bind to the surface of calcite crystal and are found in ACC and calcite, [3,4] calcium carbonate prepared via a solution method as in the current case have been reported to not possess hydroxyl ions.[5] Further, prior ptychographic tomography studies of calcite crystals of different sizes and prepared using different methods have consistently reported an electron density of 0.79 e $Å^{-3}$.[2,6] No significant spatial variations were detected, with inner and outer regions of calcite crystals exhibiting the same reduced electron density. Since vacancy formation is generally less sensitive to crystal growth parameters than hydroxyl or water incorporation, we consider vacancies the more likely cause, though minor incorporation of hydroxyl ions or water and cannot be ruled out. The formation of localized, spatially resolved, regions of even further reduced electron density or pores at 500°C in calcite crystals (Figure 4), similar observed in transmission electron micrographs of ACC post solid state crystallisation (Figure S16), does not aid clarification in our opinion. Their formation can be explained by the migration and coalescence of vacancies, and the release of trapped species (e.g., CO_2 and H_2O), leading to a local lattice collapse and a corresponding reduction in electron density.

Supplementary Note 2: Partial Volume Effects and Finite Size of Calcite Crystals Formed via Solid-State Crystallization of Amorphous Calcium Carbonate

Upon heating to 500 °C, large, spatially resolved calcite crystals become apparent in the in situ PXCT data. However, many calcite domains produced by the solid-state crystallization of dehydrated amorphous calcium carbonate (ACC) remain at or below the estimated spatial resolution of the tomograms (S11). Such small crystal sizes arise because mass transport through the loosely connected network of ACC particles is limited, and because multiple nucleation/ crystal growth fronts within ACC aggregates compete for the same material, capping the growth rate of individual crystals. As these sub-resolution calcite particles form and ripen during both the heating ramp and the subsequent isothermal hold at 500 °C, the electron-density histogram (Figure 2) develops an asymmetric baseline spanning 0 to 0.79 $e^- Å^{-3}$. Voxels at ~0.79 $e^- Å^{-3}$ represent fully resolved calcite, whereas voxels near zero correspond to macroscopic pores in the sampled volume. Instead of the baseline shifting toward higher densities with time/ temperature, it rises toward lower densities, an initially counterintuitive trend. This behaviour can be understood by considering how multiple low-density dry ACC voxels (~0.19 $e^- Å^{-3}$) reorganize into single high-density calcite voxel (~0.79 $e^- Å^{-3}$). For example, converting four ACC voxels into one calcite voxel frees three voxels' worth of volume, which become pores and boost the low-density count in the histogram. Intermediate densities (0–0.79 $e^- Å^{-3}$) arise from partial-volume effects, where a single voxel contains both calcite and void; because the measured electron density is a volume-weighted average, these mixed voxels produce a continuous gradient in the histogram rather than distinct peaks. Importantly, the increasing baseline asymmetry of this intermediate region during the isothermal hold at 500 °C, albeit slowly, indicates that Ostwald-type ripening does not cease entirely but proceeds under limited mass-transport conditions. Thus, the increasing slope of the histogram toward lower densities provides a clear, quantitative indication of ongoing crystal growth throughout the entire high-temperature period, despite the spatial resolution limits of the tomographic measurement.

Supplementary Note 3: Crystallization Behaviour of Vaterite Microspheres

The in situ PXCT measurement also captured the solid-state transformation of a small number of polycrystalline vaterite spheres already present in the as-synthesized material. These micron-sized particles exhibit a broad density range, centring at 0.68 e^{-} $Å^{-3}$. At 25 °C, both inter-particle and intra-particle density variations are observed. While some spheres have internal densities as low as 0.49 e^{-} $Å^{-3}$, their outer shells reach up to 0.76 e^{-} $Å^{-3}$, matching the theoretical density of vaterite. Zoning and density variations arise from internal porosity and the incipient transformation to calcite.[7] Upon heating to 250 °C, even initially homogenous vaterite spheres (Figure S14a&b)[7,8] develop an internal texture, i.e. a slight reduction in core density and densification at the periphery. Continued heating to 500 °C accelerates this, producing a fibrous interior and shell consisting of nanosized calcite crystals (Movies S5&6). Interestingly, the presence of a heterogenous nucleant, in the form of larger calcite crystal in contact with a vaterite sphere (Figure 13c&d), has only a limited effect on its solid-state recrystallization behaviour. While the calcite crystal grows modestly and its facets sharpen upon heating, feeding from the vaterite, numerous smaller calcite nuclei also emerge throughout the vaterite sphere. This suggests that diffusion-limited growth of the larger crystal cannot relieve the local supersaturation generated within the vaterite matrix upon heating, leading instead to “homogeneous” nucleation of new calcite domains.

Supplementary Fig. 1: Fourier-Transform Infra-Red Spectra and Transmission Electron Micrograph of the As-Synthesized ACC. (a) FTIR spectra of the as-synthesized ACC (ID0), and the three anhydrous polymorphs of calcium carbonate (Aragonite, Calcite and Vaterite).[9] Polymorph-specific or fingerprint bands are highlighted. A comparison with reference spectra reveals the synthesis of hydrated ACC, as evidenced by the broad band at 3300 cm^{-1} indicative of its hydrated state and the broad central band at 1402 cm^{-1}. (b) Transmission electron micrograph of the as synthesised ACC. The micrograph is consistent with the tomography observations, showing spherical ACC particles, particle aggregates and intra-particle/ aggregate gaps and channels. Source data are provided as a Source Data file.

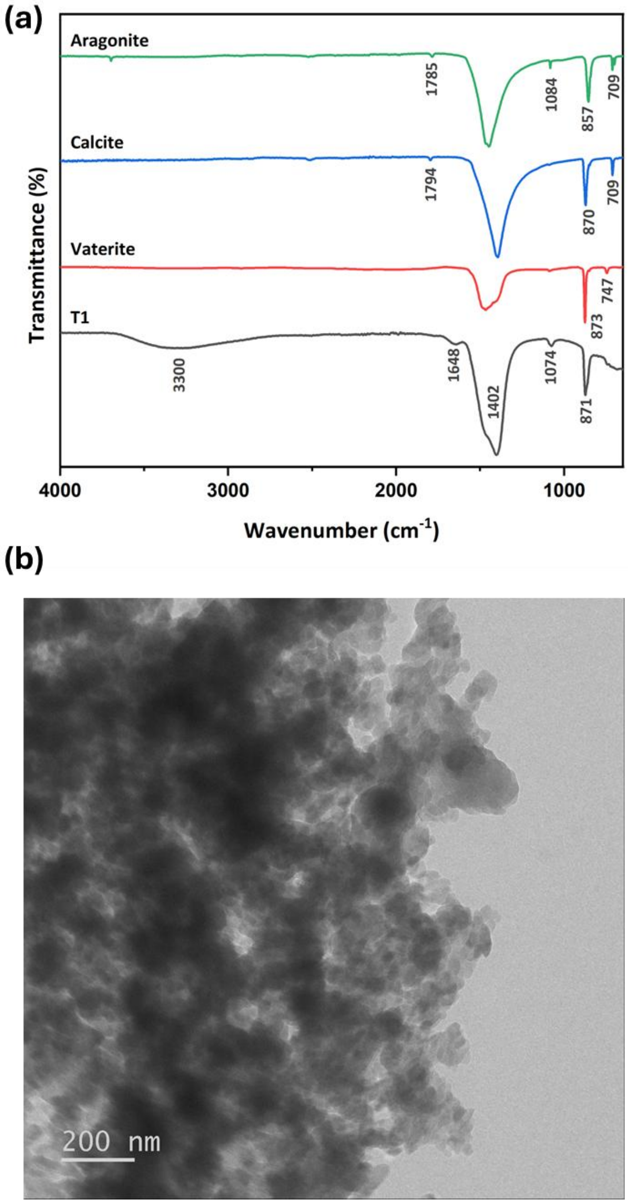

Supplementary Fig. 2: Optical Micrograph of a Tapered Silica Capillary Loaded with ACC Particles. The orange box shows the approximate field-of-view of the PXCT measurements.

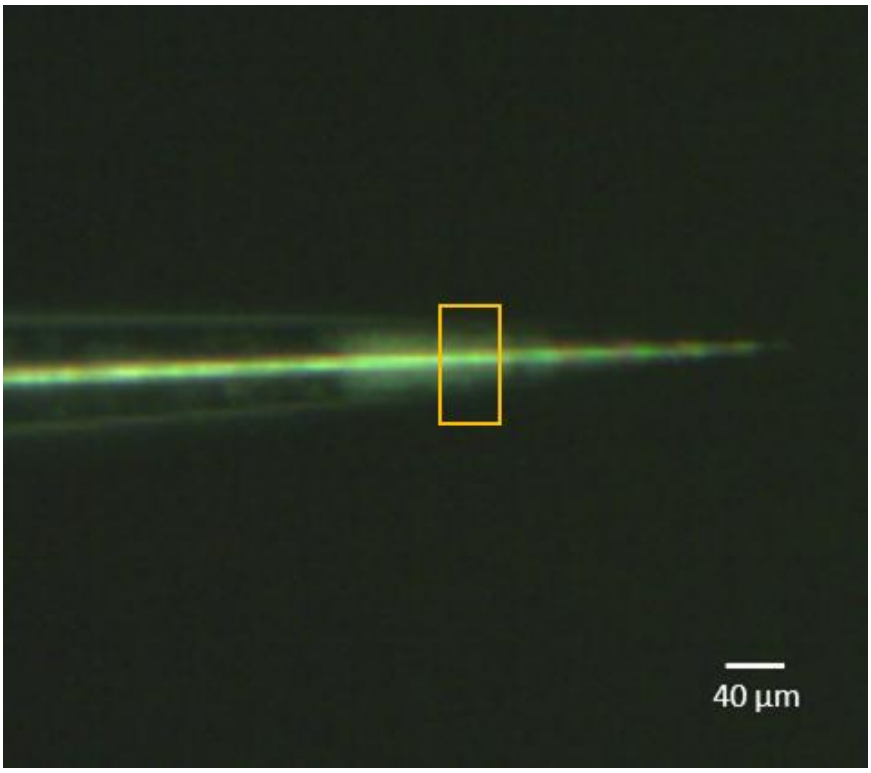

Supplementary Fig. 3: Photograph of the In Situ PXCT Setup at the cSAXS Beamline of the SLS. Instrumentation details including schematics are provided in Holler et al. (2022).[10]

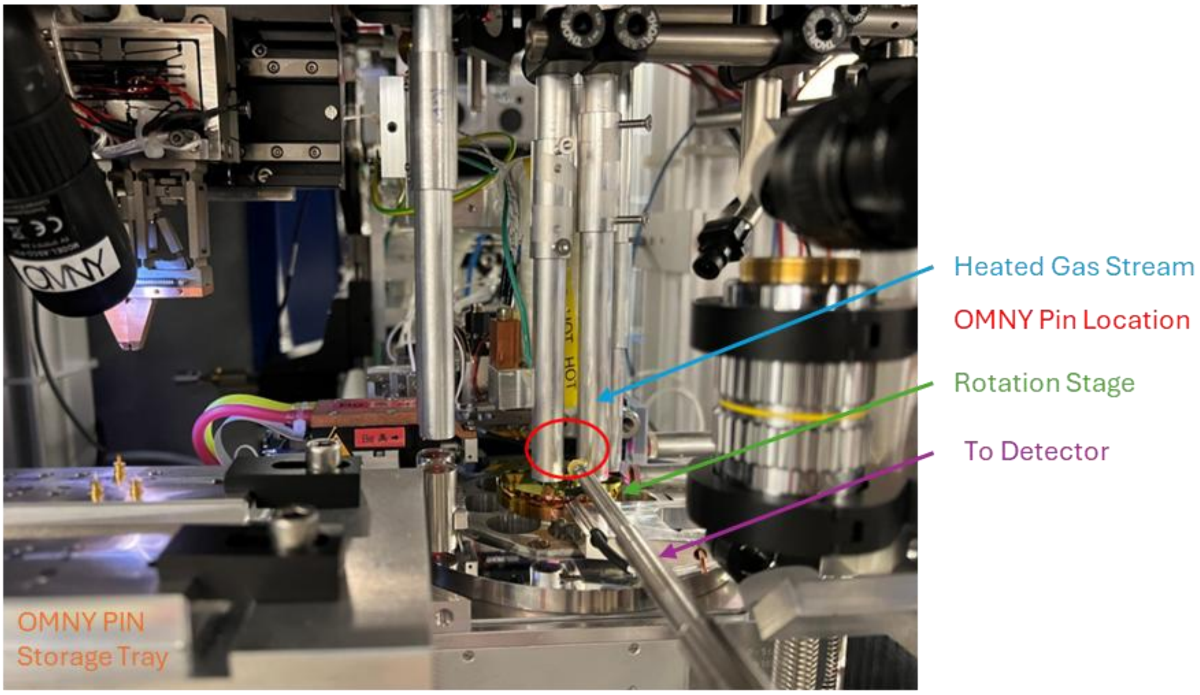

Supplementary Fig. 4: Spatial Resolution of Ptychographic Image Reconstructions. Fourier ring correlation (FRC) curve of two independently acquired and reconstructed phase contrast projections of the sample. The projections were acquired at the same rotation angle. The resolution is given by the intersection of the correlation curve with the 1-bit threshold. The pixel size is 82.84 nm and the estimated spatial resolution is 113.45 nm. Source data are provided as a Source Data file.

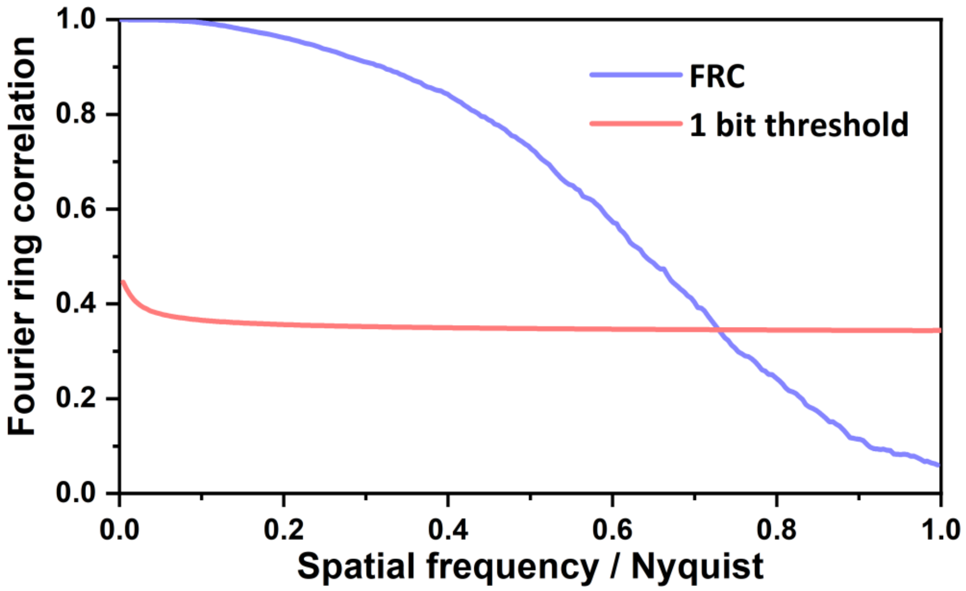

Supplementary Fig. 5: **Spatial Resolution of Electron Density Tomograms Obtained via Dynamic Tomographic Reconstruction**. Shown are Fourier shell correlation (FSC) curves for four electron density tomograms obtained via dynamic tomographic reconstruction. Each tomogram corresponds to a time point randomly selected from one of the four investigated temperature ranges. The spatial resolution estimate is defined by the intersection of the correlation curves with the ½-bit threshold curve. The spatial resolution estimates for the tomograms with ID 19, 94, 195 and 295 are 151.29, 110.29, 112.00 and 151.29 nm, respectively. The voxel size is 82.84 nm. Source data are provided as a Source Data file.

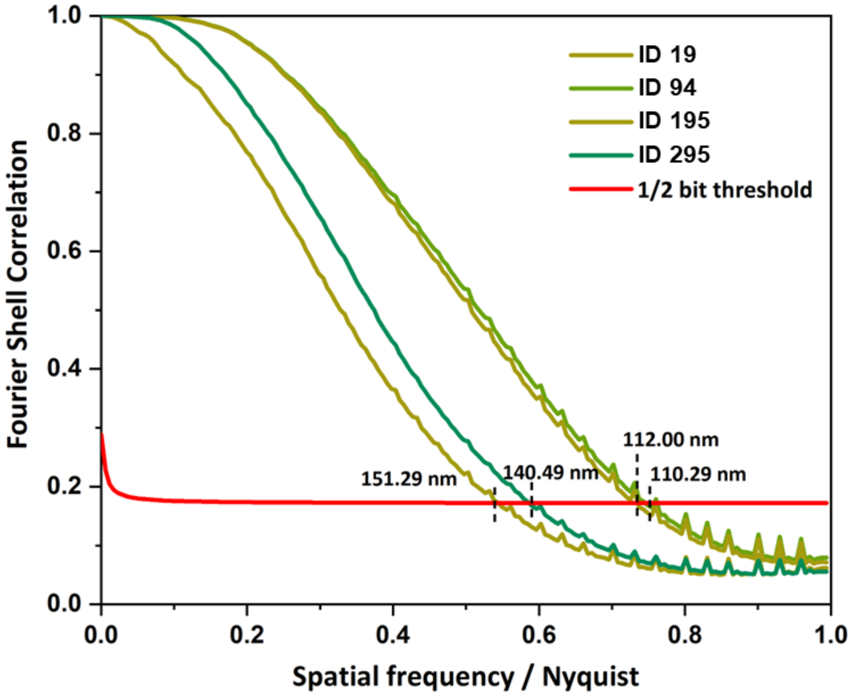

Supplementary Fig. 6: **Estimate of Temporal Resolution of Dynamically Reconstructed Tomograms.** To assess the temporal resolution of the dynamic tomographic reconstruction, the full projection dataset was split into two interleaved subsets. Each subset was independently reconstructed using the step-function model, which assigns a transition time to each voxel based on changes in electron density. Plotted below is the distribution of the difference in transition times (T_{error}) between the two reconstructions for each voxel. The full width at half maximum (FWHM) of this distribution was selected here to obtain a first pessimistic estimate of the temporal resolution or accuracy of the dynamical tomogram reconstruction. The approach captures uncertainty in assigning a transition point for example, in view of finite temporal sampling and projection noise. Source data are provided as a Source Data file.

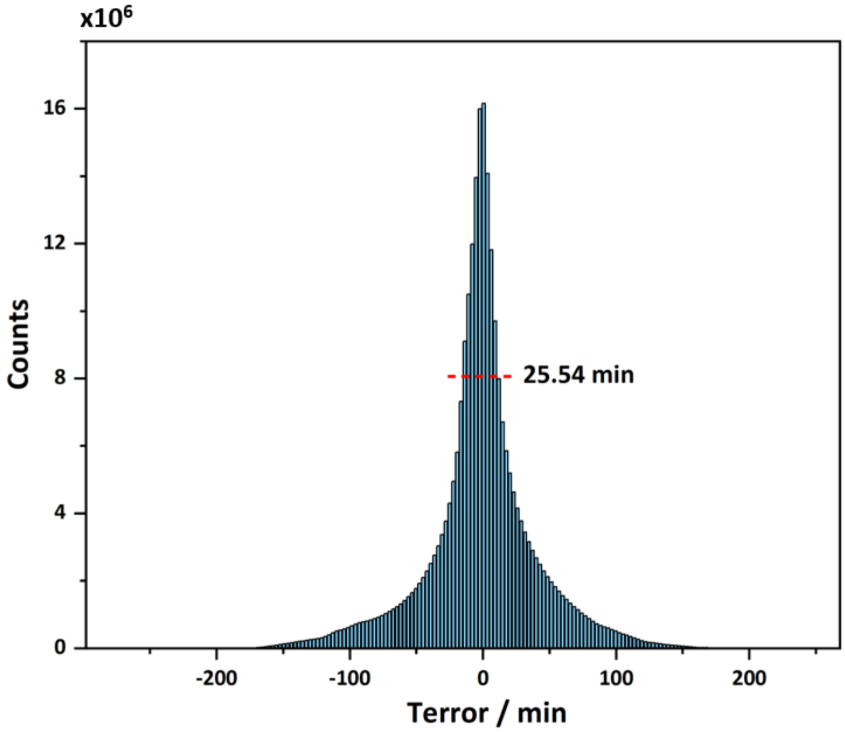

Supplementary Fig. 7: Sample Mass as a Function of Temperature. Plotted are the weight loss profiles of hydrated ACC as a function of temperature, measured by in situ PXCT and bulk thermogravimetric analysis (TGA), alongside a differential scanning calorimetry (DSC) trace. These bulk measurements were performed to confirm the consistency between the tomographic environment and standard bulk measurement conditions. TGA and DSC were conducted under continuous heating. The DSC trace identifies key thermal events, including the loss of physisorbed and structural water, solid-state crystallization, and decomposition into calcium oxide above 550 °C. The PXCT-derived mass loss closely matches the bulk TGA profile, with a minor offset of 1–2% that persists beyond 250 °C. The offset is likely due to slight differences in the initial water content. Notably, no significant weight loss was observed in the in-PXCT data even following a prolonged hold temperature at 500°C, confirming the absence of oxidative reactions or a decomposition to calcium oxide and carbon dioxide. The overall agreement validates both the temperature calibration and the environmental fidelity of the in situ PXCT setup. Source data are provided as a Source Data file.

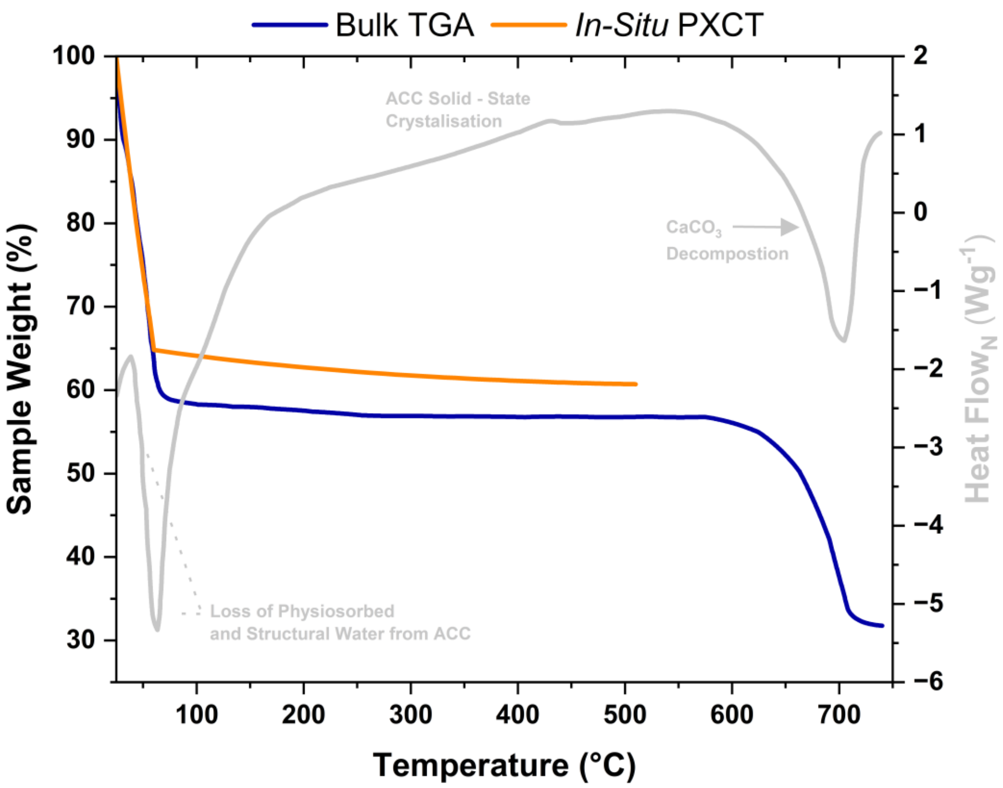

Supplementary Fig. 8: Sample Morphology as a Function of Temperature. Shown are a series of scanning electron micrographs of ACC as a function of temperature. A transition occurs from (loosely packed) hydrated ACC (25°C), to dehydrated and more densely packed ACC particles (40 & 250°C) and their eventual solid-state crystallization (500°C). Scale bars are 1 µm. The morphological changes associated with solid-state crystallization are comparatively subtle, as expected given the limited mass-transport length scales and the absence of a fluid phase. Rather than large-scale faceting or particle rearrangement, crystallization proceeds locally within the confined particle aggregates. This results in a partial loosening of the initially dense packing and the formation of larger pores within aggregate interiors, consistent with multiple crystallization centres competing for adjacent material to sustain growth. The resulting morphology visually resembles a coarsening or Ostwald-ripening-like redistribution of material, in agreement with the in situ PXCT observations (Figure 2, Movie S1, Note S2). The orange dot in the SEM images highlights a calcite crystal with a similar morphology and appearance of that shown in Figure 4 of the main text. Multiple crystals of this are found in the sample population.

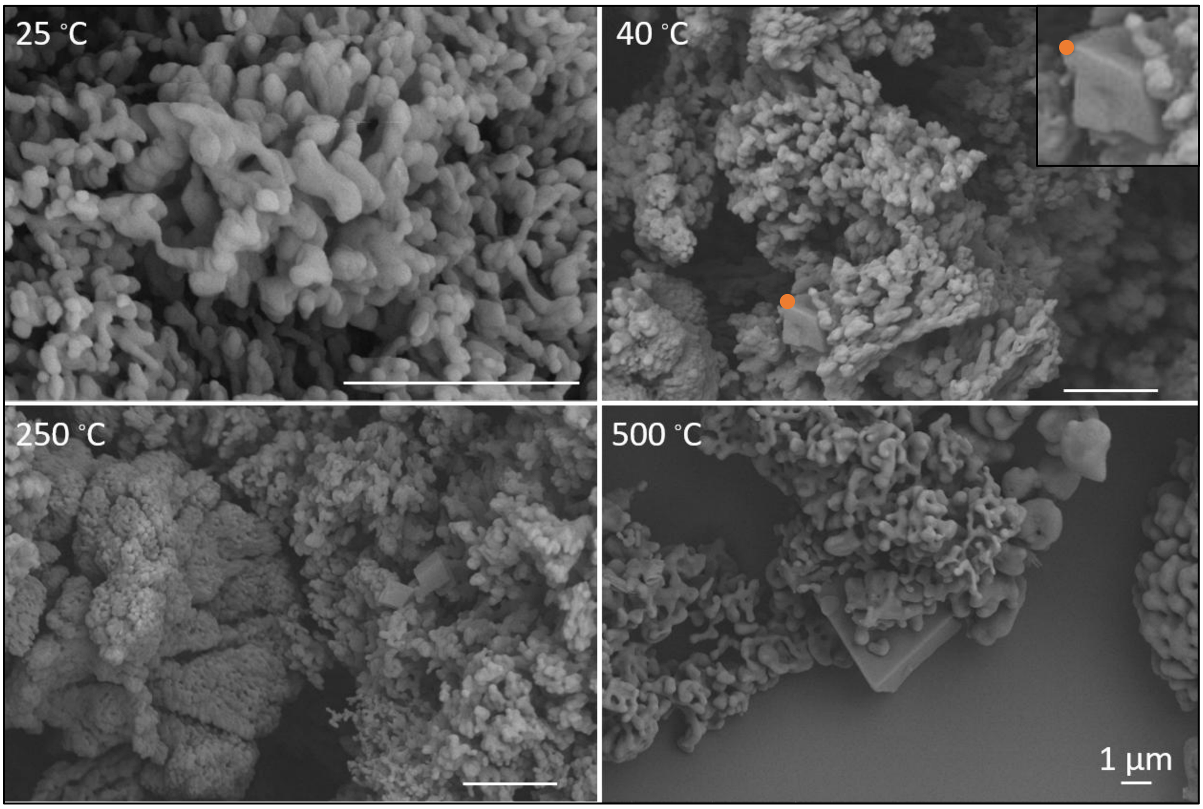

Supplementary Fig. 9: Ex-situ FTIR Spectra and PXRD pattern of ACC Prior and Post Heating. (a) Ex-situ FTIR spectra of the prepared ACC at 25°C and after heating to 500°C, demonstrating the transformation from hydrated amorphous calcium carbonate (ACC) to calcite. Characteristic carbonate group vibrations are observed at ν_3 (~1,402 cm^{-1}), ν_1 (~1,075 cm^{-1}), and ν_2 (~871 cm^{-1}). Water-related bands at ~1,648 cm^{-1} and at ~3,300 cm^{-1}, indicative of surface and structural water, confirm the hydrated nature of the initial ACC sample.[39] The absence of polymorph indicators, such as the ν_4 bands at ~742 cm^{-1} (vaterite) and ~710 cm^{-1} (calcite), further confirms the amorphous nature of the as synthesized ACC on the bulk level. Upon heating to 500°C, the water bands disappear, and the emergence of distinct calcite bands confirms the solid-state crystallization. (b) Powder X-ray diffraction (PXRD) patterns of the as-synthesized ACC before and after heating. The initial ACC sample exhibits a broad diffraction feature at ~31° 2θ, characteristic of amorphous calcium carbonate, along with a peak at ~22° 2θ originating from the silica sample holder. Heating to 500°C results in the appearance of sharp diffraction peaks characteristic of crystalline calcite, further confirming the crystallisation into calcite. Intensity is reported in arbitrary units (a.u.). Source data are provided as a Source Data file.

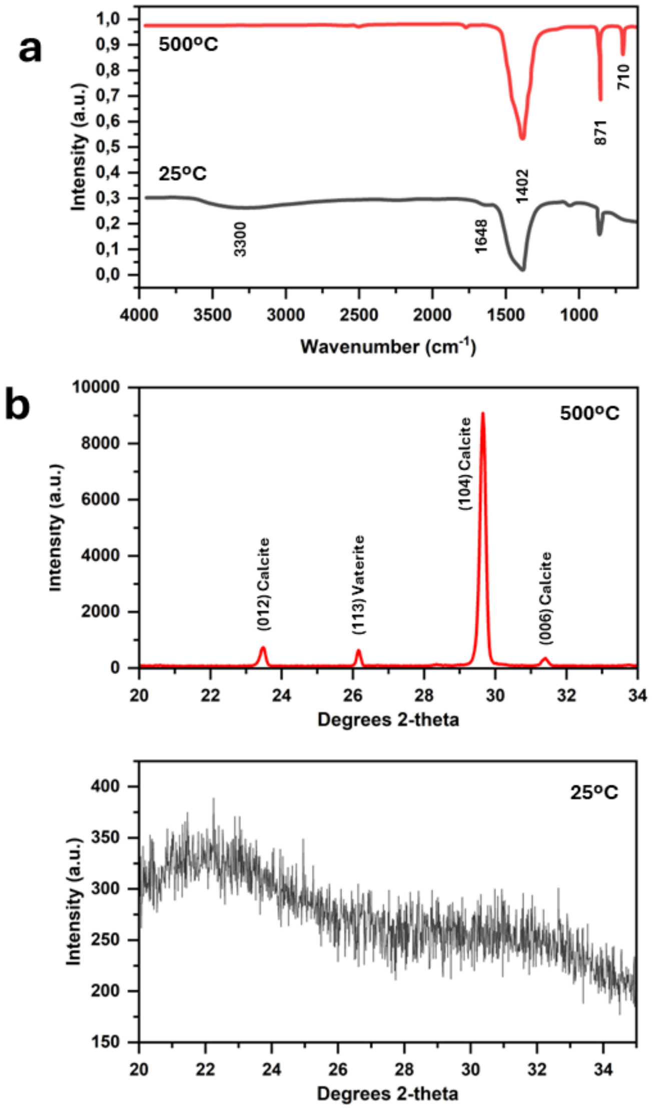

Supplementary Fig. 10: Comparison Between Dynamically Reconstructed and Filtered Back-Projection Tomograms. Example orthoslices at four different timepoints/ temperatures through: (a) the dynamically reconstructed 4D tomogram and four individually reconstructed tomograms obtained using regular filtered back-projection. Each filtered back-projection reconstruction was based on 200 projections acquired at the specified temperature. The orthoslices highlight the metastable phase (orange dot) as a function of temperature, which is clearly visible regardless of reconstruction approach. Compared to conventional filtered back-projection reconstructions, the presented dynamic sparse tomography method achieves higher spatial resolution, as more local structure and details can be observed. It also provides much higher temporal resolution as can be seen in Figure 4, being able to track not only the occurrence but also the formation and crystallization of the metastable polymorph in detail.

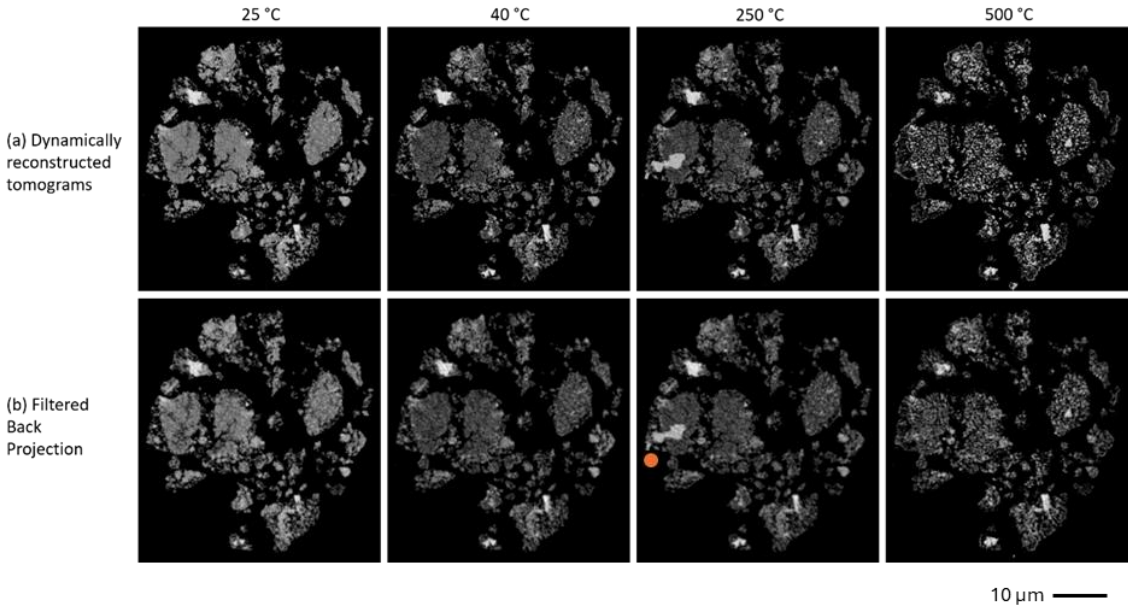

Supplementary Fig. 11: Local Electron-Density Evolution of an ACC Particle Aggregate. (a) Shown are orthoslices through an isolated aggregate of ACC particles at 25 °C, 40 °C, 250 °C, and 500 °C. (b) Voxel-wise electron-density difference maps calculated between successive temperature steps. From 25 °C to 40 °C, a pronounced electron-density loss (≈ 0.25 $e^{-}Å^{-3}$) occurs predominantly at the aggregate periphery, consistent with dehydration. Between 40 °C and 250 °C, electron-density loss remains localized to the outer regions but is substantially reduced (≈ 0.13 $e^{-}Å^{-3}$). Upon heating to 500 °C, a clear electron-density increase (≈ 0.25–0.30 $e^{-}Å^{-3}$) emerges within the aggregate core, accompanied by a corresponding depletion in surrounding regions, indicating localized solid-state crystallization and internal mass redistribution rather than continued dehydration.

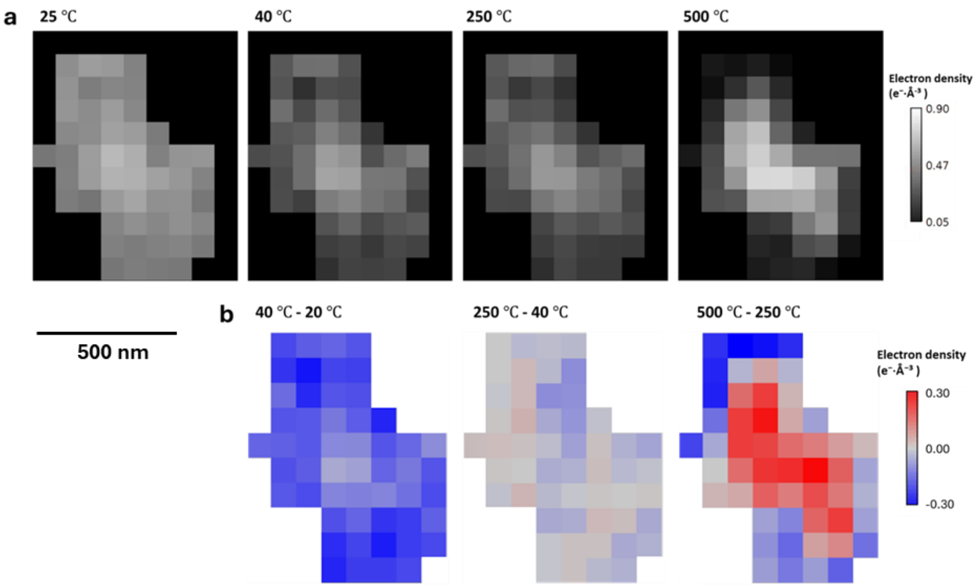

Supplementary Fig. 12: Correlation Between Initial Electron Density of a Voxel and its Crystallization Likelihood. (a) Initial electron density distribution of voxels containing amorphous calcium carbonate (ACC) that crystallize into calcite. The analysis is segmented into three temperature ranges, 25–40°C (black), 40–250°C (red), and 250–500°C (blue), to differentiate between solution-mediated and solid-state crystallization. (b & c) Selected orthoslices illustrating the spatial distribution of crystallizing ACC particles: (b) 25–40 °C; (c) 250–500 °C. The data indicates that regardless of temperature, partially or fully dehydrated ACC preferentially serves as nucleation or crystallization centre. In solution-mediated crystallization, nucleation tends to initiate at the exterior of the ACC aggregates, while crystallization occurs more uniformly throughout the aggregates at higher temperatures. The colour bar in (b) and (c) represents the initial electron density of each voxel. Voxels containing pre-existing calcite or vaterite, as well as any metastable crystals, were excluded from the analysis using a threshold cutoff of >0.55 e⁻ Å⁻³. Source data are provided as a Source Data file.

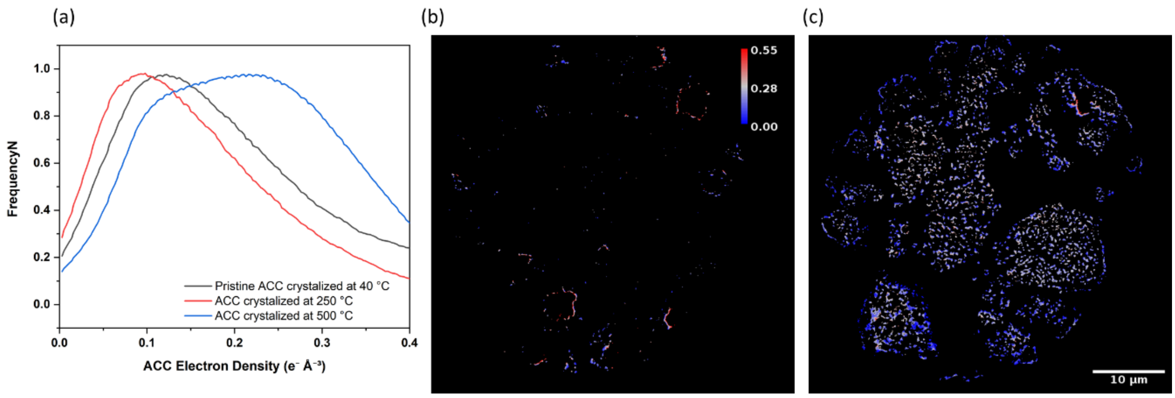

Supplementary Fig. 13: Voxel-Level Principal Component Analysis to Determine Reoccurring Crystallization Pattern. Principal component analysis (PCA) was applied at the voxel level to identify recurring electron-density patterns as a function of time / temperature. Each component represents a characteristic pattern that recurs across spatially separated regions of the sample. (a) Within-cluster sum of squares (WCSS) as a function of the number of components (k = 1–12). Based on the WCSS trend and subsequent visual inspection, these twelve components were found to dominantly describe the 4D dataset and the therein contained crystallization patterns. (b) Component-averaged electron-density evolution as a function of temperature/time for the twelve components. Components are ordered from top to bottom and grouped by physical association for clarity. Assignment to specific transformation pathways is based on the starting and final electron densities, and the shape of the density evolution. The top group shows density profiles associated with pre-existing calcite and calcite formation via ACC dissolution and recrystallization at low temperature (dashed). A gradual density decrease at elevated temperature reflects the release of trapped water and impurities (see also Figure 4). The following group corresponds to pre-existing vaterite, plotted is the dissolution of vaterite voxels, and a population recrystallizing to calcite (Figure S14). Notably, voxels undergoing recrystallization exhibit a higher initial electron density. Subsequent components represent hydrated ACC populations, we distinguished between voxels undergoing solid-state crystallization from those being consumed during this process (Figure 2a & S13). The next group captures dry and partially dehydrated ACC populations. The bottom group corresponds to the formation, dissolution, and recrystallization of metastable intermediate phases (Figure 3). Deviations of component densities from ideal/ theoretical values arises from partial-volume effects, local compositional heterogeneity, and in-component averaging. Relative volumetric contributions of each component are shown in Figure 2d. Source data are provided as a Source Data file. (c) Top: Orthoslices extracted from the electron-density tomograms. An orthoslice is shown for each 25 °C, 60 °C, 250 °C, and 500 °C, revealing the evolution of the sample with increasing temperature. Shown is the same sample height. Bottom: The same orthoslices showing the spatial distribution of selected PCA components at the same temperature. For clarity, only a subset of components is displayed, these are: pre-existing calcite (red), hydrated ACC (purple), dry or partially dehydrated ACC (cyan), and the formation–dissolution of a metastable intermediate phase (bright pink).

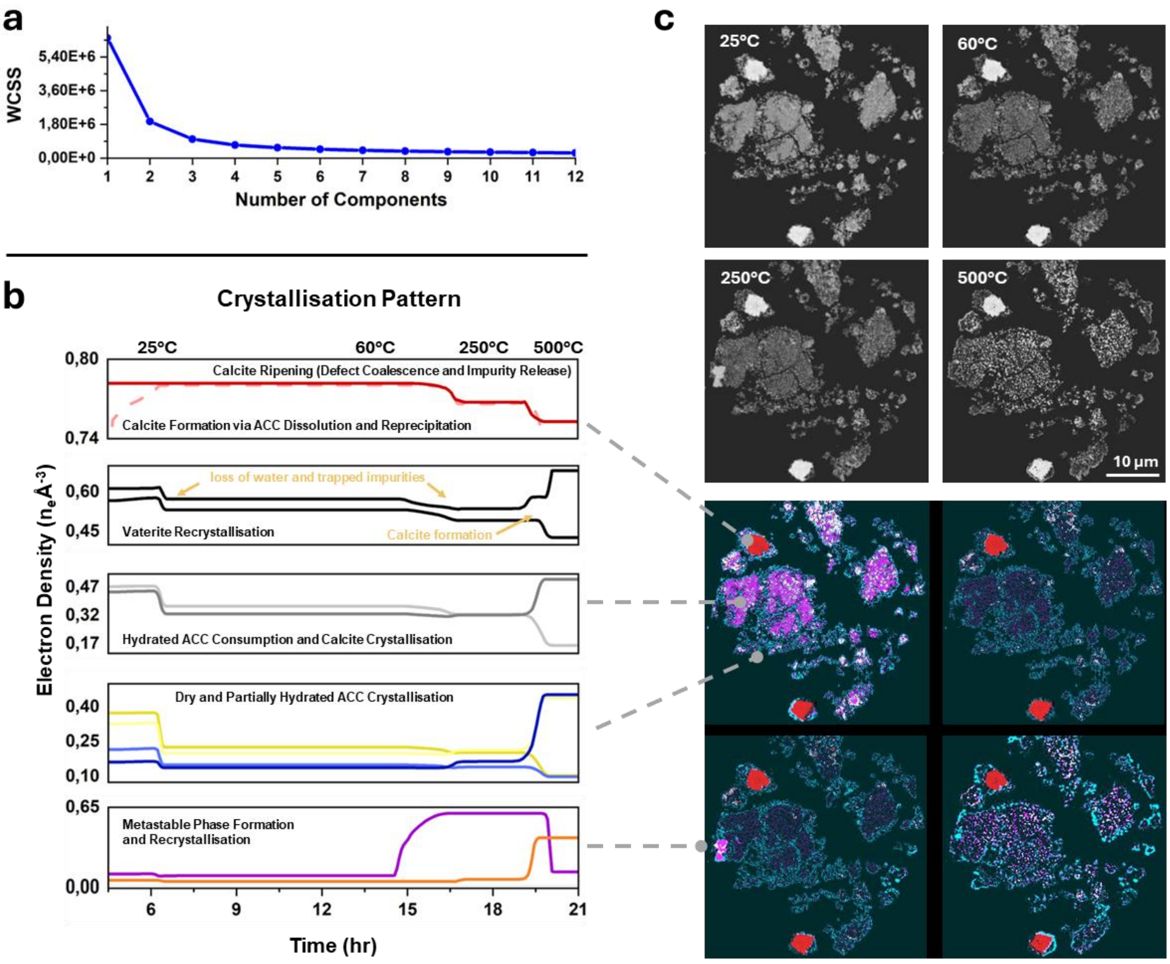
a
WCSS
5,40E+6
3,60E+6
1,80E+6
0,00E+0
1 2 3 4 5 6 7 8 9 10 11 12
Number of Components
b
Crystallisation Pattern
25°C
60°C
250°C
500°C
0,80
0,74
Calcite Ripening (Defect Coalescence and Impurity Release)
Calcite Formation via ACC Dissolution and Reprecipitation
0,60
0,45
loss of water and trapped impurities
Vaterite Recrystallisation
Calcite formation
0,47
0,32
0,17
Hydrated ACC Consumption and Calcite Crystallisation
Dry and Partially Hydrated ACC Crystallisation
0,40
0,25
0,10
0,65
0,00
Metastable Phase Formation
and Recrystallisation
Electron Density ($n_e Å^{-3}$)
6 9 12 15 18 21
Time (hr)
c
25°C
60°C
250°C
500°C
10 µm

Supplementary Fig. 14: Solid-State Crystallisation Behaviour of Vaterite. (a) Volume renderings of a polycrystalline vaterite microsphere undergoing solid-state crystallisation to calcite with increasing temperature. The emergence of macroscopic pores (violet) and calcite (red) is shown within the semi-transparent envelope of the original vaterite sphere. Scale bar, 1 μm. (b) Local electron density histograms and radial density profiles highlighting the spatial aspect of the crystallisation process. Source data are provided as a Source Data file. (c) Volume renderings of an adjoining pair of a vaterite microsphere and calcite crystal across a range of temperatures, stressing the influence of a heterogeneous crystallization interface. Scale bar, 1 μm. (d) Local electron density histograms and line profiles corresponding to the paired interface highlighted in (c). Source data are provided as a Source Data file.

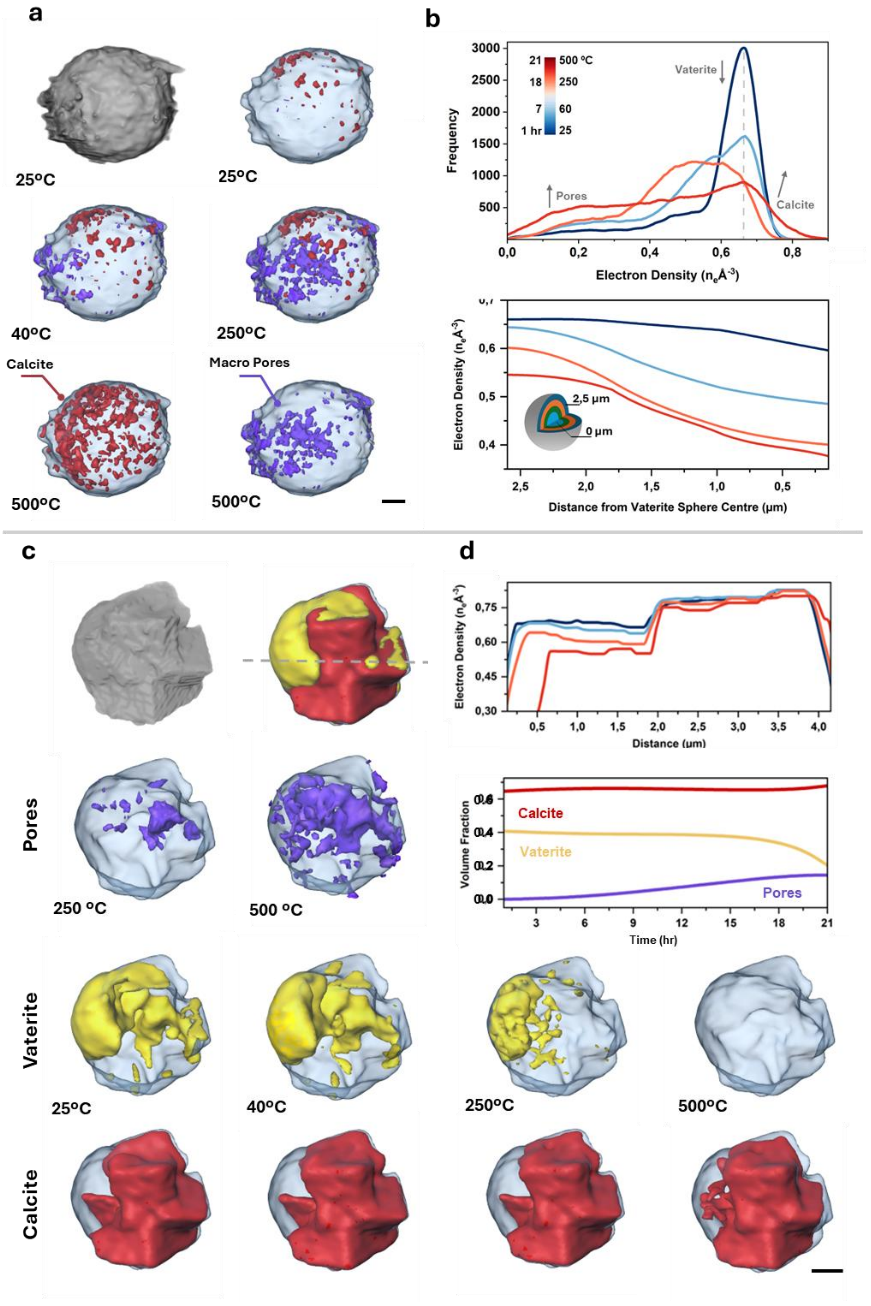

Supplementary Fig. 15: Electron Density Histogram Prior to Masking of the Silica Capillary and Surrounding Air. Shown is the histogram of the pristine-state tomogram, before masking voxels corresponding to air (inside and around the capillary) and the silica capillary itself. The capillary was segmented using a combination of thresholding and morphological operations. Source data are provided as a Source Data file.

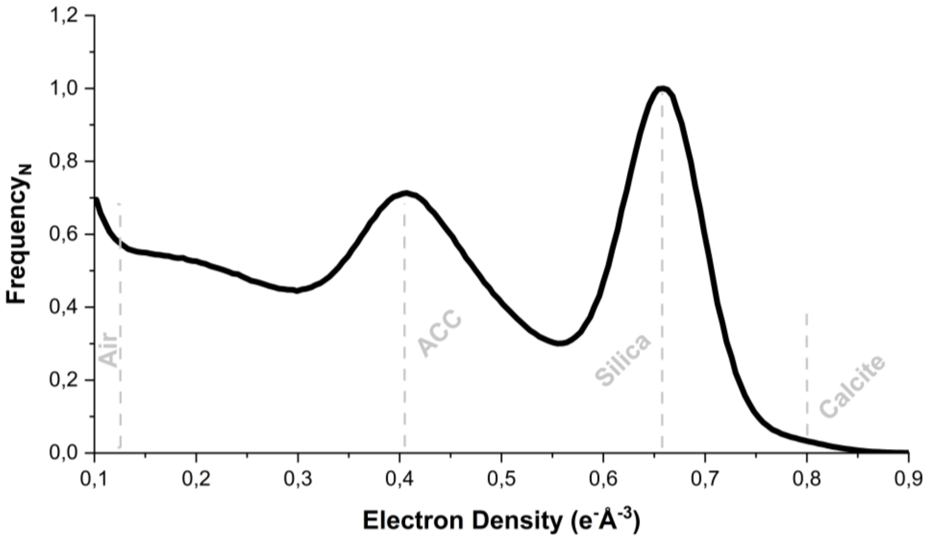

Supplementary Fig 16: Transmission Electron Micrograph of Calcite formed via Solid-State Crystallization of ACC at 500ºC in Air. Visible is the formation of nanopores upon heating.

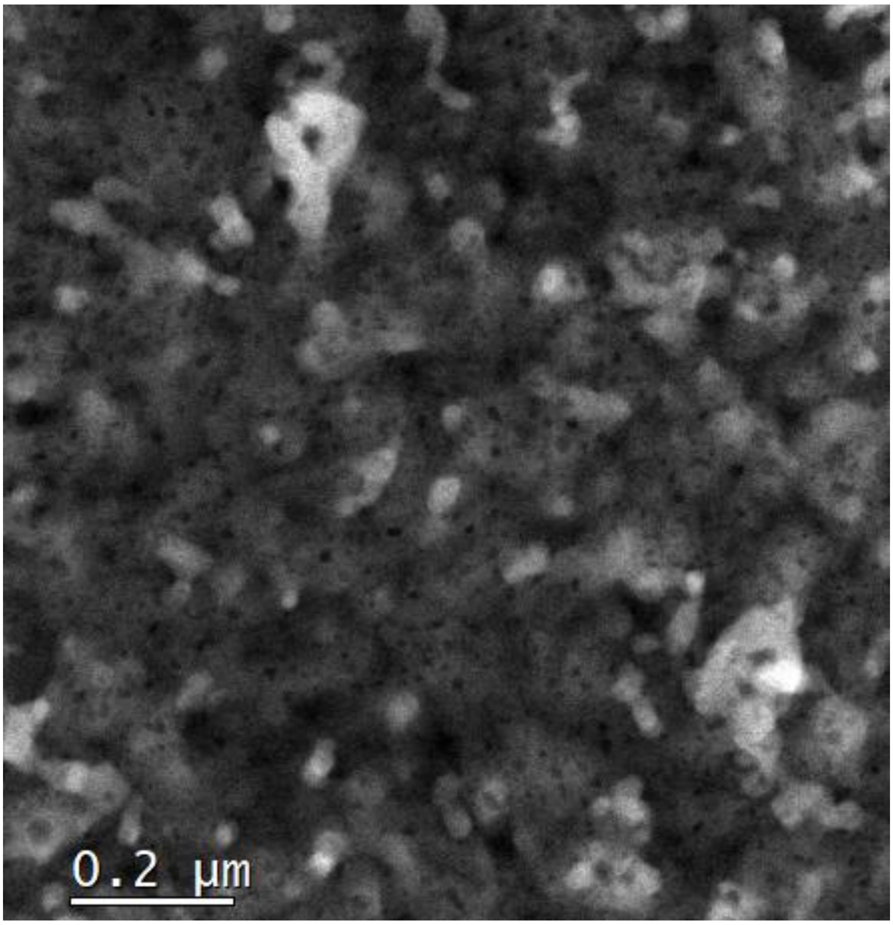

Supplemental Table 1. Electron Densities of Known Sample Components, ACC and $CaCO_3$ Polymorphs.

Components	Mass density (g/cm^3)	Total electron number (e)	Molar mass (g/mol)	Electron density ($e/Å^3$)
Air				0.0
Water	0.99	10	18	0.33
Fused SiO_2	2.1	30	56	0.67
Dry ACC ($CaCO_3$)	**0.63***	50	100	**0.19***
Partially Hydrated ACC ($CaCO_3$:xH_2O)	-§	-	-	**0.24***
Hydrated ACC ($CaCO_3$:H_2O)	1.55	60	118	**0.47***
Mono-hydrocalcite ($CaCO_3$: H_2O)	2.08	60	118	0.60
Hemihydrate $CaCO_3$ ($CaCO_3$:1/2H_2O)	2.22	55	109	0.67
Vaterite	2.54	50	100	0.76
Calcite	*2.71*	*50*	*100*	0.82

Calcite Polymorphs not expected to form under the experimental conditions or detected in control experiments.

Ikaite ($CaCO_3$: $6H_2O$)	1.77-1.93	110	208	0.56-0.61
Aragonite	2.93	50	100	0.88

*****This work. §The physical density of partially hydrated ACC, which contains only structural water, can currently not be determined due to potential convolution between packing density and hydration level, both determining the measured electron density of partially hydrated ACC. Assuming a hydration level of 10% ($CaCO_3 \cdot 0.1H_2O$), the mass density is estimated to be ~0.8 g/cm^3.

Supplementary References

1 Ihli, J. *et al.* Visualization of the effect of additives on the nanostructures of individual bio-inspired calcite crystals. *Chemical Science* **10**, 1176–1185 (2019). https://doi.org/10.1039/C8SC03733G

2 Ihli, J. *et al.* Ptychographic X-ray tomography reveals additive zoning in nanocomposite single crystals. *Chemical Science* **11**, 355–363 (2020). https://doi.org/10.1039/C9SC04670D

3 Schmidt, M. P., Ilott, A. J., Phillips, B. L. & Reeder, R. J. Structural Changes upon Dehydration of Amorphous Calcium Carbonate. *Crystal Growth & Design* **14**, 938–951 (2014). https://doi.org/10.1021/cg401073n

4 Kim, Y.-Y. *et al.* Hydroxyl-rich macromolecules enable the bio-inspired synthesis of single crystal nanocomposites. *Nature Communications* **10**, 5682 (2019). https://doi.org/10.1038/s41467-019-13422-9

5 Kuriyavar, S. I. *et al.* Insights into the formation of hydroxyl ions in calcium carbonate: temperature dependent FTIR and molecular modelling studies. *Journal of Materials Chemistry* **10**, 1835–1840 (2000). https://doi.org/10.1039/B001837F

6 Shirani, S. *et al.* X-ray near-field ptychographic nanoimaging of cement pastes. *Cement and Concrete Research* **185**, 107622 (2024). https://doi.org/https://doi.org/10.1016/j.cemconres.2024.107622

7 Trushina, D. B., Bukreeva, T. V., Kovalchuk, M. V. & Antipina, M. N. $CaCO_3$ vaterite microparticles for biomedical and personal care applications. *Materials Science and Engineering: C* **45**, 644–658 (2014). https://doi.org/https://doi.org/10.1016/j.msec.2014.04.050

8 Christy, A. G. A Review of the Structures of Vaterite: The Impossible, the Possible, and the Likely. *Crystal Growth & Design* **17**, 3567–3578 (2017). https://doi.org/10.1021/acs.cgd.7b00481

9 Ihli, J. *et al.* Dehydration and crystallization of amorphous calcium carbonate in solution and in air. *Nature Communications* **5**, 3169 (2014). https://doi.org/10.1038/ncomms4169

10 Holler, M. *et al.* Environmental control for X-ray nanotomography. *Journal of Synchrotron Radiation* **29**, 1223–1231 (2022). https://doi.org/doi:10.1107/S1600577522006968
