# Supplementary material for: In situ ptychographic x-ray nanotomography of temperature-controlled crystallization processes
Source: Nat Commun. 2026 May 29;17:6994. doi: 10.1038/s41467-026-73738-1 (PMC13392228; doi:10.1038/s41467-026-73738-1)
Supplement: Supplementary file 2 — Description of Additional Supplementary Files [file 41467_2026_73738_MOESM2_ESM.pdf]

Description of Additional Supplementary Files

File Name: Supplementary Movie 1

Description: Animated orthoslice through the centre of the sample volume, showing the sample's evolution as a function of time and temperature.

File Name: Supplementary Movie 2

Description: Animated volume rendering of the formation of a calcium carbonate hemihydrate crystal.

File Name: Supplementary Movie 3

Description: Animated volume rendering of the recrystallisation of the same calcium carbonate hemihydrate crystal into calcite.

File Name: Supplementary Movie 4

Description: Animated volume rendering of a volume defect-rich calcite crystal as a function of temperature.

File Name: Supplementary Movie 5

Description: Animated volume rendering highlighting the crystallization of a micron-sized vaterite sphere into calcite as a function of temperature.

File Name: Supplementary Movie 6

Description: Animated volume rendering showing the crystallization of a vaterite sphere in contact with a calcite crystal as a function of temperature.
