# Supplementary material for: In situ ptychographic x-ray nanotomography of temperature-controlled crystallization processes
Source: Nat Commun. 2026 May 29;17:6994. doi: 10.1038/s41467-026-73738-1 (PMC13392228; doi:10.1038/s41467-026-73738-1)
Supplement: Supplementary file 9 — Transparent Peer Review file [file 41467_2026_73738_MOESM9_ESM.pdf]

nature portfolio

Peer Review File

In Situ Ptychographic X-ray Nanotomography of Temperature-Controlled Crystallization Processes

Corresponding Author: Dr Johannes Ihli

This file contains all reviewer reports in order by version, followed by all author rebuttals in order by version.

Version 0:

Reviewer comments:

Reviewer #1

(Remarks to the Author)
This manuscript presents a technically impressive and scientifically impactful demonstration of dynamic in situ ptychographic X-ray nanotomography applied to the crystallization of amorphous calcium carbonate (ACC) under controlled temperature conditions. The authors successfully visualize both dominant and rare transformation events, including the identification of a metastable hemihydrate polymorph and the nanoscale defect evolution during thermal coarsening. This study showcases the potential of the method as a versatile platform for probing dynamic materials behavior in complex environments.
I believe the manuscript makes a strong contribution and should be accepted after minor revisions.

The following points should be addressed or clarified in a revised version:
1. Justification for the use of ptychography
While the reported spatial resolution (~130 nm) is excellent, it is worth noting that comparable resolution and phase contrast can also be achieved by full-field imaging X-ray microscopes using Fresnel zone plates and phase plates. The authors should more explicitly highlight what specific advantages ptychography offers in this context—e.g., quantitative electron density mapping, greater depth of field, reduced optical aberrations, or better performance under in situ conditions (e.g., beamline flexibility, sample geometry, etc.).
2. Sample oxidation
The manuscript mentions thermal dehydration and crystallization processes, but does not discuss whether oxidation of the sample (especially at 500°C in open capillaries) might affect the structure or chemistry. A brief discussion or clarification on whether oxidation effects are negligible under the reported experimental conditions would improve clarity.
3. Thermal drift of the sample
High-resolution in situ imaging at elevated temperatures is typically vulnerable to sample drift or mechanical instability. The authors should clarify whether thermal drift was observed, and if so, how it was corrected or mitigated during acquisition and reconstruction.
4. Temperature limitations of the current setup
The custom in situ system is reported to support heating up to 850°C. The current study goes up to 500°C. Can the authors comment on the feasibility and limitations of operating at temperatures above 800°C using their platform? Would thermal expansion, radiation damage, or X-ray optics impose practical constraints?
5. Current limitations and future perspectives
It would strengthen the paper to briefly reflect on the current limitations of the technique—such as scan speed, radiation dose, reconstruction time, or material compatibility—and outline how these might be addressed in the near future (e.g., higher frame rates, AI-assisted reconstruction, advanced X-ray optics, multi-modal integration, etc.).

Overall, I strongly support publication of this work after minor revision. The method developed is highly relevant and broadly applicable across a range of material science and environmental systems, and the findings are novel and clearly presented.

(Remarks on code availability)

Reviewer #2

(Remarks to the Author)
The paper by Jiang et al reports the use of x-ray nanotomography to visualize structural changes in amorphous calcium carbonate during heating. A powder of ACC is heated stepwise to 500 degrees while continuously rotated and imaged over 25 hours. After reconstruction of ptychographic projections, a dynamic tomographic reconstruction is done which produces superior results (time/space resolution) compared to filtered back projection. Per voxel electron densities are calculated from the reconstructions and used to analyze the composition and its changes throughout the measurement. The authors claim the reconstructions show a few phase transitions, dehydration and crystallization, and also the appearance and disappearance of meta-stable phases.

Main comments:
Overall this is a nice demonstration of a dynamic tomography measurement. My main concern is that while there is strong evidence for the dehydration of ACC there is not equally strong evidence for the crystallization to calcite during the measurement. I think the most compelling evidence are the PCA patterns rather than the histograms provided in the main text. The end result histogram show lots of pore space and perhaps some calcite (figures 2B and 3C). However, it's hard to visually interpret the relationship between the PCA patterns in Figure S12 with the per voxel changes. For example, in Figure S12B, the middle pattern shows two curves labelled "Hydrated ACC Consumption and Calcite Crystallization". One trends down at the end and the other trends up. Is that pore space and calcite crystal volume? What does that actually look like in the volume? It's not possible to tell from the images given. Why are those densities so different from those in the top pattern (Calcite formation via ACC dissolution…)?

The ex situ diffraction measurements do seem to clearly show the crystallization. But ACC is unstable to there's no guarantee that the sample used in the ptychography measurements saw the same transition. It would be nice to see clear zoomed in regions showing the local change from lower to higher density. As presented, it looks primarily like loss of density due to dehydration.

Other comments:
Line 315: change "Rate" to "Rare"

Line 351-352: ethanol water mixtures are known to stabilize ACC. ACC is unstable in air with a lifetime to crystallization of a couple of days. What was the time between synthesis and measurement? More detail regarding handling should be given.

Line 920: the quoted spatial resolution of 113 nm is less than twice the pixel size which is physically impossible (i.e. you cannot resolve 113 nm objects with 82 nm pixels). The best possible spatial resolution is about 165 nm.

Line 65: states that they achieve "nanometer spatial and minute-scale temporal resolution". In reality they estimate ~110 nm spatial resolution and 25 minute temporal resolution so I think the statement is inaccurate. I would call this "sub-micron" and "sub-hour" resolution.

Line 244: after the statement about poor resolution in previous works they cite a paper with higher resolution than that which is presented here, reference 65. It also has 10X higher time resolution.

Lines 63,96,284,285,377: The paper says near field ptychography but it really looks like far field ptychography with a zone plate. Can they explain how the near field measurements were made? It looks like they're measuring diffraction patterns rather than holograms. And there is no diffuser (that I can tell) which is required by near-field setups.

Lines 481-483: they should add more information about uncertainty. What is the local uncertainty in volumes that they are analyzing and assigning composition to? For example, the meta-stable phase.

Lines 420-429: The analysis of the speed increase is incomplete. Certainly ptychography doesn't require 250000 times fewer photons than conventional scanning microscopy. So their estimate of the time for scanning probe is way too high since it shouldn't require 50ms per point. Any gain is mostly due to a slow scanning system which is just an engineering problem and not inherent to the imaging methods. I think the claim here should be softened.

Line 221-222: PXCT is not detecting defects, as fluorescence or diffraction would. PXCT has no direct sensitivity to the defects and their presence is rather guessed based on electron density which depends on many factors. This statement should be clarified.

line 55: change "statistical" to "statistically"
line 86: change "transformation" to "transformations"

(Remarks on code availability)

Reviewer #3

(Remarks to the Author)
The authors present in their manuscript an in-situ monitoring of the crystallization process of the amorphous calcium

carbonate (ACC) while heating up to 500 degrees C using near-field ptychography. Their measurements are followed by extensive data processing, reconstruction, and analysis, visualizing the different stages of crystallization. While the results are impressive – especially the combination of in-situ heating and the demonstrated spatial resolution – the manuscript lacks clarity in several aspects and requires clarification in a major revision.
Major concern: The focus of the manuscript remains unclear.
Is the crystallization process and its in-situ tracking over the temperature the key novelty? In this case, the manuscript should contain a clear motivation for focusing on the crystallization of amorphous calcium carbonate and why it is essential to visualize the different crystallization stages.
Is the focus much more on the imaging method? In this case, the focus must be sharpened to stress the impressive performance of the near-field ptychographic nanotomography in these in-situ experiments. I recommend not emphasizing the uniqueness in achieving the specifications, like the high temporal (minute-scale) and spatial (nanoscale) resolution, allowing for representative volume sizes. A closer look at the reported temporal resolution (5-25 minutes per tomogram), spatial resolution (130 nanometers), and the analyzed sample size (diameter 80 micrometersl) reveals that other nano-tomography techniques like for example, nearfield-holotomography or X-ray Zernike phase-contrast nanotomography, can achieve similar performance at the same or even larger sample sizes in similarly or even shorter times (see https://doi.org/10.1107/S1600577522012103 or https://doi.org/10.1364/BOE.396695). In-situ heating in combination with nanoscale imaging has already been demonstrated https://doi.org/10.1016/j.ceramint.2023.11.216. The in-situ study with near-field ptychographic nanotomography at this speed and resolution is the apparent novelty. The advantages of using near-field ptychography to study the crystallization processes (e.g., accurate electron density mapping, resolving minimal changes in materials) should be emphasized more clearly. Also, more details are required on the choice of the amorphous calcium carbonate and why it is essential to visualize the different crystallization steps.
Minor details:
• Lines 115-116: The cited publication on the speed of analytical reconstruction methods is from 1970. The authors should cite a more recent work on tomographic reconstruction and compare the speed of their approach with the state-of-the-art tomographic reconstruction.
• Abstract and line 165: The term ‘Ostwald ripening’ needs an explanation. Please do not use special terms without introducing them.
• Lines 481ff: Electron Density Uncertainty is estimated by the standard deviation of the electron density of the surrounding air. This is only a good measure for the electron density uncertainty in a static process. In the dynamic process (heating and crystallization), the uncertainty of one voxel within the specimen is larger than that of the non-changing voxel with surrounding air. The voxels are averaging for 25 minutes over a changing sample region. This should increase the uncertainty.
• Lines 521ff, Voxel-level Analysis: The authors should explain why a Principal Component Analysis allows for identifying the dominant crystallization patterns. This might not be clear to every reader.

(Remarks on code availability)

Version 1:

Reviewer comments:

Reviewer #1

(Remarks to the Author)
I thank the authors for their thoughtful and comprehensive revisions of the manuscript entitled "In Situ Ptychographic X-ray Nanotomography of Temperature-Controlled Crystallization Processes".
All of my previous comments have been addressed thoroughly and satisfactorily. I recommend acceptance of the manuscript in its current form.

(Remarks on code availability)

Reviewer #2

(Remarks to the Author)
The authors have addressed all of my concerns.

(Remarks on code availability)

Reviewer #3

(Remarks to the Author)
The authors carefully revised their manuscript, significantly improving its clarity. I am fully satisfied with the authors' answers to all reviewers' comments and with their changes to the manuscript. I have no further comments on the manuscript and recommend it for publication.

(Remarks on code availability)

Open Access This Peer Review File is licensed under a Creative Commons Attribution 4.0 International License, which permits use, sharing, adaptation, distribution and reproduction in any medium or format, as long as you give appropriate credit to the original author(s) and the source, provide a link to the Creative Commons license, and indicate if changes were made.
In cases where reviewers are anonymous, credit should be given to 'Anonymous Referee' and the source.
The images or other third party material in this Peer Review File are included in the article's Creative Commons license, unless indicated otherwise in a credit line to the material. If material is not included in the article's Creative Commons license and your intended use is not permitted by statutory regulation or exceeds the permitted use, you will need to obtain permission directly from the copyright holder.
To view a copy of this license, visit https://creativecommons.org/licenses/by/4.0/

Manuscript NCOMMS-25-54379-T2024 - Response to Referees

We thank the referees and editorial staff for their detailed, constructive, and positive reviews of our manuscript, which have led to significant improvements.

As suggested, we have expanded the manuscript to more clearly highlight the advantages of ptychography, and reflect on current limitations and possible future developments of the presented approach. We have also sharpened the focus of the manuscript by addressing several technical and scientific points raised in the reviews, including but not limited to sample oxidation, near-field ptychography, and measurement uncertainty. In addition, in response to Reviewer 2's concerns regarding the crystallization step, we have added the requested Figure and provided further explanation on the PCA analysis and the interpretation of the presented data.

Provided below is a point-by-point response to the referees' comments and editorial requests. The comments are presented in black, the responses in blue. Changes to the manuscript are quoted below the respective replies. Changes in the manuscript file are highlighted in yellow.

REVIEWER COMMENTS

Reviewer #1 (Remarks to the Author):

This manuscript presents a technically impressive and scientifically impactful demonstration of dynamic in situ ptychographic X-ray nanotomography applied to the crystallization of amorphous calcium carbonate (ACC) under controlled temperature conditions. The authors successfully visualize both dominant and rare transformation events, including the identification of a metastable hemihydrate polymorph and the nanoscale defect evolution during thermal coarsening. This study showcases the potential of the method as a versatile platform for probing dynamic materials behavior in complex environments.

I believe the manuscript makes a strong contribution and should be accepted after minor revisions.

The following points should be addressed or clarified in a revised version:

1. Justification for the use of ptychography

While the reported spatial resolution (~130 nm) is excellent, it is worth noting that comparable resolution and phase contrast can also be achieved by full-field imaging X-ray microscopes using Fresnel zone plates and phase plates. The authors should more explicitly highlight what specific advantages ptychography offers in this context—e.g., quantitative electron density mapping, greater depth of field, reduced optical aberrations, or better performance under in situ conditions (e.g., beamline flexibility, sample geometry, etc.).

We thank the reviewer for the suggestion. We have expanded the introduction, adding a section on full-field X-ray nanotomography and ptychographic tomography highlighting the advantages it offers and its strategic position. The corresponding section of the introduction reads as follows:

"Bulk techniques, including spectroscopy and scattering, provide statistically representative results but cannot resolve spatial dependencies and often fail to capture rare and or transient events.[21-24] *Full-field micro-X-ray tomography has become a central tool for in situ and operando studies due to its ability to rapidly examine millimetre-sized samples at micrometre resolution and its straightforward integration with diverse sample environments.*[3,7,25,26] *By contrast, full-field in situ X-ray nanotomography, which enables rapid examination of micrometre-sized samples at tens to hundreds of nanometres spatial resolution, remains comparatively rare.*[26-32] *Achieving such resolutions typically relies on nanofocusing optics, which introduce trade-offs between field of view and spatial resolution, limit depth of field, and impose tight spatial constraints, complicating the integration of environmental control systems and iterative nanoposition corrections needed for extended measurements under varied conditions. Scanning nanoprobe X-ray tomography offers even higher spatial resolution, but long acquisition times, and similar stringent optical and stability requirements, have largely restricted studies to ex situ conditions and small volumes.*[33] *X-ray ptychography and its tomographic extension, ptychographic X-ray computed tomography (PXCT),*[34-36] *are scanning, lensless, coherent diffraction imaging techniques that are maturing into general user methods at synchrotron facilities. PXCT enables the acquisition of quantitative electron-density tomograms of extended sample volumes with spatial resolution limited not by X-ray optics but by the recovered scattering signal and available coherent flux, and can, in principle, approach wavelength-limited resolution. This allows the use of micrometre-scale illumination probes while retaining nanometre spatial resolution and quantitative contrast across extended sample volumes, providing practical advantages such as an effectively extended depth of field, robustness against optical aberrations, high dose efficiency, and flexibility in sample geometry and experimental design. However, because ptychography yet remains a scanning technique, tomographic implementations have so far been largely restricted to ex situ studies due*

to long acquisition times and the lack of integrated environmental control and sample-positioning/correction instrumentation.

Here, we present an integrated platform for in-situ dynamic ptychographic X-ray nanotomography that combines …"

2. Sample oxidation

The manuscript mentions thermal dehydration and crystallization processes, but does not discuss whether oxidation of the sample (especially at 500°C in open capillaries) might affect the structure or chemistry. A brief discussion or clarification on whether oxidation effects are negligible under the reported experimental conditions would improve clarity.

A clarification of possible oxidation effects and the thermal decomposition pathway was added.

Calcium carbonate (calcite and related carbonate phases) does not undergo oxidation under the experimental conditions applied here. Instead, its principal high-temperature pathway is thermal decomposition (decarbonation) into CaO and CO_2, which begins slightly below 600 °C, thus above the maximum temperature used in this study. This decomposition process is illustrated in Figure S7. As shown by both the TGA and PXCT data, the mass loss observed up to 500 °C can be attributed entirely to the release of water and trapped impurities. The continuous hold in the PXCT experiment at 500 °C shows no further mass loss, confirming that no oxidative reactions occur and that the setup does not exceed the specified temperature within the stated error, i.e. not thermal decomposition occurs. We added the following section to the results section after discussing the tomography evident sample changes on heating to 500°C.

"At 250 and 500 °C, the calcite peak sharpens, and the histogram baseline gradually rises, which is consistent with the formation of calcite crystals and ongoing Ostwald ripening. …. Notably, no significant changes in sample mass were observed during the observation period at 500 °C (Figure S7), indicating that the observed structural evolution can be fully attributed to crystallization processes, with no evidence for oxidative or thermal decomposition of the $CaCO_3$*"*

Further, we added the following to the caption of Figure S7:

"The PXCT-derived mass loss closely matches the bulk TGA profile, with a minor offset of 1–2% that persists beyond 250 °C, likely due to slight differences in the initial water content. Notably, no significant weight loss was observed in the in-PXCT data even following a prolonged hold at 500°C, confirming the absence of oxidative reactions or a decomposition to calcium oxide and carbon dioxide."

3. Thermal drift of the sample

High-resolution in situ imaging at elevated temperatures is typically vulnerable to sample drift or mechanical instability. The authors should clarify whether thermal drift was observed, and if so, how it was corrected or mitigated during acquisition and reconstruction.

The reviewer is correct that sample and instrument drift/ instability are typically the dominant factors limiting in-situ nanotomography at elevated temperatures and or across extended periods. Drift directly deteriorates spatial resolution and often requires repeated manual re-centering and alignment between tomograms/ projections, making the individual tomogram acquisition speed less relevant if substantial realignment steps are needed. This challenge is a key reason why high-resolution studies across a broad temperature range and over extended periods remain scarce.

In our case, the developed *in situ* PXCT setup minimizes drift by mechanically decoupling the heat source from the interferometer-corrected, actively cooled sample position/ correction stage. This configuration ensures sub-10 nm sample stability across both broad environmental conditions and extended experimental periods. Drift, specifically with regards to the sample's center of rotation, does essentially not occur as the temperature of the reference mirror of the interferometer system is actively stabilized to the room temperature. Further position refinements applied during post-acquisition tomographic alignment, do not differ from the procedures applied otherwise at room temperature. While horizontal sample drifts are dominantly minimized by the flex-structure of the cooled sample stage, the residual vertical expansion of the sample carrying tomography pin, resulted in a drift or reduced the effective tomographic field of view compared to the projection dimensions over the 500 degree window, i.e. to a common sample region present in all projections regardless of temperature. The main text and Methods section have been updated to clarify these points. Instrumentation details are presented in detail in the cited instrument reference.

Changes in the main text:

*In situ ptychographic nanotomography was performed under controlled conditions using a custom-built positioning instrument equipped with an environmental control system (**Figure 1a**). This setup allows selective sample heating up to 850 °C and control over the sample surrounding gas atmosphere composition, while maintaining 360° rotational freedom and sub–10 nm sample positioning accuracy; critically, over extended durations and across the full temperature range.*[38] *The commonly spatial-resolution and field-of-view deteriorating sample drift is minimized by mechanically decoupling the heat source from the actively cooled, interferometer-corrected sample positioning stage.*

We further added the following to the corresponding method section:

"To ensure positional stability during in situ heating, the environmental PXCT setup mechanically decouples the heat source from the actively cooled and interferometer-corrected sample positioning instrument.[38,88] *This design minimizes thermally induced drift, providing sub–10 nm stability throughout data acquisition up to 850 °C. Any residual lateral drift of the sample's centre of rotation is continuously monitored and corrected in real time by the interferometer feedback system, with additional refinement during tomographic alignment. Vertical drift, primarily due to thermal expansion of the OMNY pin carrying the sample, remains unavoidable to a certain extent. This can be compensated by shifting the vertical field-of-view in dependence of temperature. A routine to automatically shift the field-of-view was not yet implemented in the current study. The fixed field-of-view applied here during projection acquisition, restricted tomographic reconstruction to a sample region/ height common to all projections across the full temperature series. As a result, the reconstructed tomograms encompassed only ~65% of the projection height. The integrated mass flow controllers of the environmental control system*[38] *maintained a steady gas flow, ensuring a stable heating profile and consistent sample temperature. The flow rate was set to 1 L min^{-1}, with temperature and humidity sensors placed approximately 2 cm from the OMNY pin tip. To facilitate continuous tomogram acquisition, a heating rate of 5°C min^{-1} was applied for each incremental temperature increase."*

4. Temperature limitations of the current setup

The custom in situ system is reported to support heating up to 850°C. The current study goes up to 500°C. Can the authors comment on the feasibility and limitations of operating at temperatures above 800°C using their platform? Would thermal expansion, radiation damage, or X-ray optics impose practical constraints?

Operation is currently possible up to ~900 °C, modifications are necessary to reach higher temperature. The main limiting element currently is the heating cartridge of the setup providing up to 165 W heating power, with which the system then reaches 900 °C. 850 °C was set as the operation-safe and experimental condition reliable upper limit. Radiative losses strongly increase when implementing a heating cartridge with more power, thus the surrounding setup has to be accordingly adapted. In addition, at temperatures above this range, several factors could compromise stable operation, including material instabilities, and increasing vertical temperature gradients across the sample. The setup maintains sample stability by mechanically decoupling the heat source from the actively cooled and interferometer-corrected sample positioning stage, allowing sub-10 nm positional stability even at high temperatures. Nevertheless, temperature gradients may develop across the sample due to the heating geometry and sample composition. At 500 °C, the sample is ~20 °C cooler than the set temperature; at 900 °C, gradients up to 50 °C may occur depending on the sample heat conductivity. Larger deviations at even higher temperatures would significantly compromise the validity of measurements. Hence the currently given upper limit. We added this information to the method section:.

"***Setup Temperature Limitation and Temperature Uncertainty:*** The developed and here utilized environmental control setup,[38] enables controlled heating of samples from room temperature up to a verified temperature of 850 °C. Although the system could in principle operate at even higher temperatures (~900 °C), the upper limit was set at 850 °C to ensure stable operation, representative experimental conditions, and to prevent potential damage to nearby beamline components. The instrument maintains sub-10 nm sample positioning stability through mechanical decoupling of the heat source from the actively cooled and interferometer-corrected sample stage. While ensuring stability, a vertical temperature gradient is unavoidable leading to a temperature-dependent deviation between the sample and the experimental set-point. The deviation is less at lower temperatures, < 5ºC under 200˚C. At 500 °C, the sample is estimated to be ~20 °C cooler than the set value, whereas at 850 °C and in view of heat transfer limitations, deviations of up to 50 °C can occur (influenced by the thermal conductivity of the sample). Operation beyond this temperature would likely introduce further uncertainty, potentially compromising measurement representativeness.

5. Current limitations and future perspectives

It would strengthen the paper to briefly reflect on the current limitations of the technique—such as scan speed, radiation dose, reconstruction time, or material compatibility—and outline how these might be addressed in the near future (e.g., higher frame rates, AI-assisted reconstruction, advanced X-ray optics, multi-modal integration, etc.).

Overall, I strongly support publication of this work after minor revision. The method developed is highly relevant and broadly applicable across a range of material science and environmental systems, and the findings are novel and clearly presented.

We thank the reviewer for the comment and the positive evaluation. The corresponding section of the discussion was rewritten and extended as follows:

"These first in situ experiments also highlight current limitations of the approach. Tomogram acquisition times and effective temporal resolution are on the order of several to tens of minutes for the here demonstrated sample volumes and spatial resolution. Both spatial and temporal resolution were dominantly limited by the coherent flux of the third-generation synchrotron source and the sample-to-detector distance, necessitating point acquisition times of tens of milliseconds and restricting scanning probe dimensions. The coherent flux of fourth-generation synchrotron sources,[72] together with larger probe sizes, is expected to open the temporal resolution bottleneck. When combined with multi-beam illumination and multi-slice reconstruction strategies,[73-75] further relaxing lateral and angular sampling requirements, these advances are projected to enable sparse tomogram acquisition times at or below the one-minute scale for the demonstrated sample volume. These developments also impact radiation dose. Although ptychography with angularly sparse sampling is already dose-efficient, increased coherent flux and extended observation times raise concerns not only for canonical considered radiation-sensitive materials, but also for fine-structured materials including integrated circuits.[76] Larger probes, relaxed sampling, and non-rigid reconstruction approaches capable of accommodating radiation-induced sample changes[40] are therefore likely to be critical. Improvements in flux, data acquisition & reconstruction, alongside detector technology, are further expected to improve spatial resolution, enabling sub-10 nm resolution for these sample volumes.

Finally, adaptive or event-driven data acquisition routines are crucially missing from the present demonstration.[77-79] In such schemes, imaging parameters including exposure time, angular sampling, field-of-view or environmental conditions such as temperature, are dynamically adjusted in response to environment-driven changes in the sample or experimental setup. Their absence, as illustrated here, can

lead to a reduced effective field-of-view or unnecessary data acquisition and radiation dose during quiescent periods, as evident in Figure 2b. Implementation of such routines will require near-real-time ptychographic image reconstruction and, ultimately, reconstruction and analysis of the evolving four-dimensional tomographic dataset. Depending on spatio-temporal resolution, volume and duration, such experiments can generate terabyte-scale datasets with estimated computational demands in the tera- to petaflop regime.[79] *Apart these HPC requirements, addressing this challenge will require automated analysis pipelines, incl. machine-learning-based methods for data reduction, and feature identification.*

Beyond improvements in spatio-temporal resolution and dose efficiency, the approach offers opportunities for functional extension. The integration of spectroscopic contrast through combined sparse X-ray energy- and angle-resolved tomographic acquisition[39,67,80-84] *would enable simultaneous tracking of structural, compositional, and chemical evolution. Collectively, these developments position in-situ ptychographic nanotomography as an evolving platform for the study of dynamic, multiscale transformations, including processes such as nucleation, spinodal decomposition, and phase-front propagation in reactive solids and liquids."*

Reviewer #2 (Remarks to the Author):

The paper by Jiang et al reports the use of x-ray nanotomography to visualize structural changes in amorphous calcium carbonate during heating. A powder of ACC is heated stepwise to 500 degrees while continuously rotated and imaged over 25 hours. After reconstruction of ptychographic projections, a dynamic tomographic reconstruction is done which produces superior results (time/space resolution) compared to filtered back projection. Per voxel electron densities are calculated from the reconstructions and used to analyze the composition and its changes throughout the measurement. The authors claim the reconstructions show a few phase transitions, dehydration and crystallization, and also the appearance and disappearance of meta-stable phases.

Main comments:

Overall this is a nice demonstration of a dynamic tomography measurement. My main concern is that while there is strong evidence for the dehydration of ACC there is not equally strong evidence for the crystallization to calcite during the measurement. I think the most compelling evidence are the PCA patterns rather than the histograms provided in the main text. The end result histogram show lots of pore space and perhaps some calcite (figures 2B and 3C). However, it's hard to visually interpret the relationship between the PCA patterns in Figure S12 with the per voxel changes. For example, in Figure S12B, the middle pattern shows two curves labelled "Hydrated ACC Consumption and Calcite Crystallization". One trends down at the end and the other trends up. Is that pore space and calcite crystal volume? What does that actually look like in the volume? It's not possible to tell from the images given. Why are those densities so different from those in the top pattern (Calcite formation via ACC dissolution…)?

The ex situ diffraction measurements do seem to clearly show the crystallization. But ACC is unstable to there's no guarantee that the sample used in the ptychography measurements saw the same transition. It would be nice to see clear zoomed in regions showing the local change from lower to higher density. As presented, it looks primarily like loss of density due to dehydration.

We thank the reviewer for this careful and constructive comments. We address the concerns regarding (i) the initial state of the sample, (ii) the evidence for solid-state crystallization during the in situ measurement, and (iii) the interpretation/ accessibility of the PCA analysis.

(i) Initial state of the sample: The concern that ACC may have partially crystallized prior to the in situ measurement is understandable, given that the preparation–measurement timeline was not explicitly stated in the original manuscript. This information has now been added (see response to R2Q1). The time between synthesis and the start of the in situ PXCT experiment was less than 30 minutes. The likelihood of crystallization during this period was further reduced by washing the freshly synthesized ACC with ethanol immediately after synthesis, and adding a 90/10 ethanol/water mixture after loading the capillary, which is known to retard crystallization. In addition, FT-IR spectra acquired directly from the sample inside the capillary immediately prior to the in situ PXCT measurement confirm the ACC state. The initial electron density measured by PXCT matches the expected value(s) for ACC, and the early-stage density evolution reflects the progressive loss of physically and chemically bound water. Together, these observations confirm that the sample entering the in situ experiment consisted predominantly of hydrated ACC. It is also well established that dehydrated ACC can remain metastable for extended periods in dry environments until sufficient thermal energy is supplied to initiate crystallization.

(ii) Evidence for crystallization during heating: The statement that the in situ data “primarily look like loss of density due to dehydration” is not correct. At temperatures above 250 °C, no sig. amount of water can physically remain in the sample regardless of whether the starting material is ACC or calcite. This is directly supported by the agreement between the in situ PXCT-derived mass loss and the ex situ TGA measurements (Supporting Figure S7). Consequently, the pronounced structural and density changes observed between 250 °C and 500 °C (see Fig 2a reproduced below) cannot be attributed to dehydration. Direct visual evidence for crystallization is apparent in the in situ data, as shown by the comparison of orthoslices at 250 °C and 500 °C in Figure 2a, Movie S1, and the PCA. These data reveal substantial changes in both morphology and local electron density, consistent with crystallisation processes.

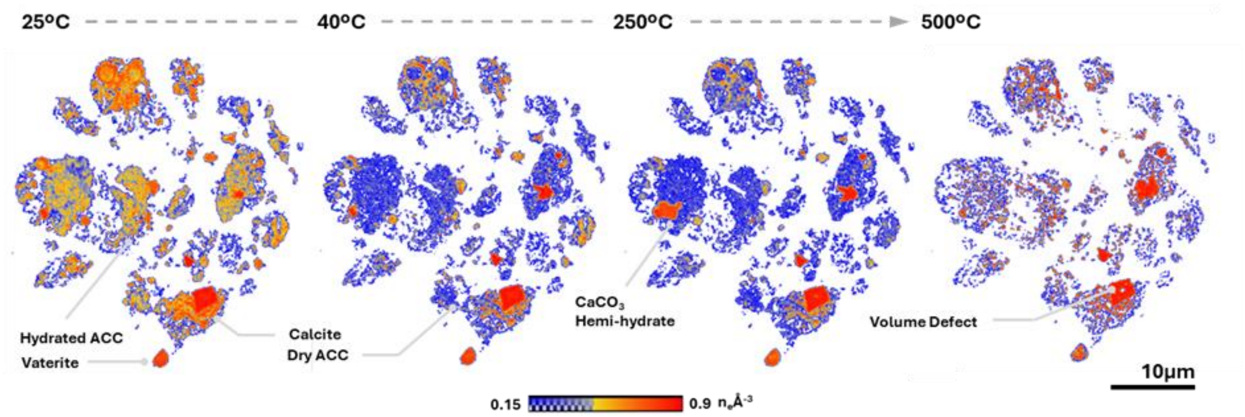

Nature of solid-state crystallization in ACC.

The expectation of large-scale densification or dramatic morphological reorganization, implicitly assumes the solution-mediated crystallization picture, and naively we expected this picture initially as well. Solid state crystalisation of loosely packed aggregates containing multiple competing nucleation and growth centers and under mass-transport-limited conditions is expected to produce spatially heterogeneous density rearrangements morphologically resembling coarsening or ripening processes. We commented on this in Note S2. To have a visually confirmation of this, we performed ex situ SEM measurements on the same batch as we did the ex-situ XRD measurements, i.e. following cross confirmation of crystalisation. Supporting Figure 8 shows the morphological evolution of ACC as a function of temperature by ex-situ SEM, we see the same morphological trends as in-situ tomograms, i.e. opening of the ACC aggregate structure. We update the Figure caption to comment on morphological evolution as well.

"Supporting Figure 8: Sample Morphology as a Function of Temperature. *…. The morphological changes associated with solid-state crystallization are comparatively subtle, as expected given the limited mass-transport length scales and the absence of a fluid phase. Rather than large-scale faceting or particle rearrangement, crystallization proceeds locally within the confined particle aggregates. This results in a partial loosening of the initially dense packing and the formation of larger pores within aggregate interiors, consistent with multiple crystallization centres competing for adjacent material to sustain growth. The resulting morphology visually resembles a coarsening or Ostwald-ripening-like redistribution of material, in agreement with the in situ PXCT observations (Figure 2, Movie S1, Note S2).*"

Local Area Zoom-In

We added the requested “zoomed-in” visualization on an isolated ACC aggregate to directly demonstrate voxel-level density increase and mass transport associated with crystallization. We show both absolute electron-density changes and voxel-wise electron-density difference maps between successive temperature steps.

“Supporting Figure 11: Local electron-density evolution of an ACC Particle Aggregate. *(a) Shown are orthoslices through an isolated aggregate of ACC particles at 25 °C, 40 °C, 250 °C, and 500 °C.* ***(b)*** *Voxel-wise electron-density difference maps calculated between successive temperature steps. From 25 °C to 40 °C, a pronounced electron-density loss (≈ -0.25 e^{-} $Å^{-3}$) occurs predominantly at the aggregate periphery, consistent with dehydration. Between 40 °C and 250 °C, electron-density loss remains localized to the outer regions but is substantially reduced (≈ -0.13 e^{-} $Å^{-3}$). Upon heating to 500 °C, a clear electron-density increase (≈ 0.25–0.30 e^{-} $Å^{-3}$) emerges within the aggregate core, accompanied by a corresponding depletion in surrounding regions, indicating localized solid-state crystallization and internal mass redistribution rather than continued dehydration.”*

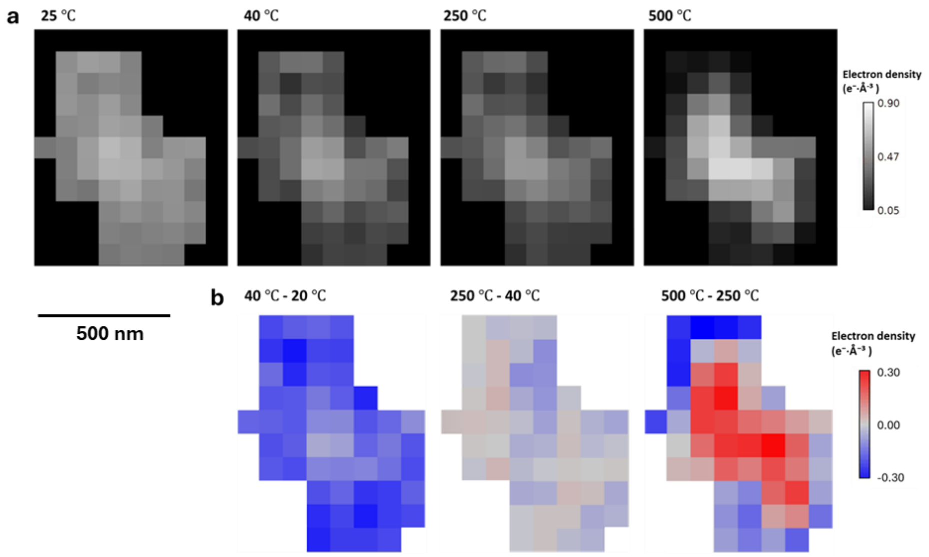

(iii) Interpretation/ accessibility of the PCA analysis. The reviewer is fully correct that the corresponding plots, Figure S12 b and c, were insufficiently explained. We added a detailed explanation to the SI figure caption to address this.

"Supporting Figure 13: Voxel-Level Principal Component Analysis to Determine Reoccurring Crystallization Pattern. *Principal component analysis (PCA) was applied at the voxel level to identify recurring electron-density patterns as a function of time / temperature. Each component represents a characteristic pattern that recurs across spatially separated regions of the sample. (a) Within-cluster sum of squares (WCSS) as a function of the number of components (k = 1–12). Based on the WCSS trend and subsequent visual inspection, these twelve components were found to dominantly describe the 4D dataset and therein contained crystallization patterns. (b) Cluster-averaged electron-density evolution as a function of temperature/time for the twelve components. Components are ordered from top to bottom and grouped by physical association for clarity. Assignment to specific transformation pathways is based on the starting and final electron densities, and the shape of the density evolution. The top group shows density profiles associated with pre-existing calcite and calcite formation via ACC dissolution and recrystallization at low temperature (dashed). A gradual density decrease at elevated temperature reflects the release of trapped water and impurities (see also Figure 4). The following group corresponds to pre-existing vaterite, plotted is the dissolution of vaterite voxels, and a population recrystallizing to calcite (Figure S14). Notably, voxels undergoing recrystallization exhibit a higher average initial density. Subsequent components represent hydrated ACC populations, distinguishing voxels undergoing solid-state crystallization from those being consumed during this process (Figure 2a & S13). The next group captures dry and partially dehydrated ACC populations. The bottom group corresponds to the formation, dissolution, and recrystallization of metastable intermediate phases (Figure 3). Deviations of component densities from ideal/ theoretical values arises from partial-volume effects, local compositional heterogeneity, and in-component averaging. Relative volumetric contributions of each component are shown in Figure 2c. (c) Top: Orthoslices extracted from the electron-density tomograms. An orthoslice is shown for each 25 °C, 60 °C, 250 °C, and 500 °C, revealing the evolution of the sample with increasing temperature. Shown is the same sample height. Bottom: The same orthoslices showing the spatial distribution of selected PCA components at the same temperature. For clarity, only a subset of components is displayed: pre-existing calcite (red), hydrated ACC (purple), dry or partially dehydrated ACC (green), and the formation–dissolution of a metastable intermediate phase (pink).“*

Other comments:

Line 315: change “Rate” to “Rare”

Thank you for spotting this, this was changed.

“Figure 3: Formation and Recrystallization of a Rare Metastable Polymorph Crystal.”

1. Line 351-352: ethanol water mixtures are known to stabilize ACC. ACC is unstable in air with a lifetime to crystallization of a couple of days. What was the time between synthesis and measurement? More detail regarding handling should be given.

This information was provided in the manuscript`s method section. However, the reviewer is correct that the explicit timeline was missing and should have been clearly stated. We apologize, the amended method section:

"…The capillary tip was left open for the solvents and hydration water to escape during the in-situ PXCT measurements. Ethanol/ water mixtures are known to stabilize ACC; the liquid was added to keep the hydration state of the ACC and prevent its crystallization during the sample transfer onto the instrument and the start of in-situ measurements. Importantly, the ACC was synthesized on-site directly prior to the sample loading and PXCT measurement. The time between synthesis and the start of in situ PXCT experiments was < 30 minutes."

2. Line 920: the quoted spatial resolution of 113 nm is less than twice the pixel size which is physically impossible (i.e. you cannot resolve 113 nm objects with 82 nm pixels). The best possible spatial resolution is about 165 nm.

We thank the reviewer for this comment. The quoted value of 113 nm refers to the correlation-based spatial resolution of the ptychographic projections, obtained from Fourier ring correlation (FRC) analysis. For tomographic datasets, the corresponding metric is Fourier shell correlation (FSC), and both FRC and FSC are standard and widely used for resolution estimation in coherent diffraction imaging and tomography. Different threshold criteria are commonly used in FRC/FSC analysis and can lead to different numerical resolution estimates, with the here used ½ and 1 bit the most common.

To avoid ambiguity, we have revised the manuscript to explicitly state (i) the voxel size of the tomograms, (ii) that the reported tomographic resolution is obtained from FSC using the half-bit information criterion, and (iii) how this relates to a classical two-pixel sampling consideration at the first main-text mention.

"These tomograms have a voxel size of 82.84 nm and a sample-averaged spatial resolution of approximately 130 nm, as determined by Fourier shell correlation analysis using the half-bit threshold criterion (Figures S4 and S5). Under the two-pixel sampling expectation, this corresponds to an average spatial resolution of ~160 nm."

3. Line 65: states that they achieve “nanometer spatial and minute-scale temporal resolution”. In reality they estimate ~110 nm spatial resolution and 25 minute temporal resolution so I think the statement is inaccurate. I would call this “sub-micron” and “sub-hour” resolution.

We thank the reviewer for this comment.

The estimated spatial resolution of ~110–130 nm (projection/tomogram) is within the nanometre range, and the temporal resolution estimate of ~25 minutes is minute-scale. As regime-level description they are accurate, and the precise numerical values (~110–130 nm, ~25 min) are stated explicitly at the beginning of the Results section, to remove any ambiguity. Estimate origins are further documented in the Methods and Supporting Information to avoid any misunderstanding.

We believe the wording is appropriate and accurate; however, we are of course happy to adjust it if the editors prefer an upper bound formulation as preferred by the reviewer.

4. Line 244: after the statement about poor resolution in previous works they cite a paper with higher resolution than that which is presented here, reference 65. It also has 10X higher time resolution.

We appreciate the comment, and are aware of the apparent conundrum at first glance.

The manuscript in question (Liu, X. *et al.*. *Nature Communications* **12**, 3441 2021) reports a voxel size of 43.34 nm. The manuscript does not report the achieved spatial resolution, instead references a previous room-temperature study reporting: "This technique has been demonstrated with a sub-50 nm 3D resolution at NSLS-II49", i.e. incomparable in both terms of resolution deteriorating sample and instrument stabilities. The study does not report a 10x higher temporal resolution. Reported is a 2-2.5 min tomogram acquisition time. In contrast, our study explicitly reports and verifies a FSC-based spatial resolution (~135 nm at the ½-bit criterion,) and a temporal resolution estimate of ~25 minutes, at 5 min acquisition. These values inherently include the effects of dose, stability and dynamic reconstruction. Required stability and resolution is retained over a 25 hour window and 500 degree range for a scanning point technique in ptychography over a larger FOV, compared to fixed temperature, ~4 hour observation window and full-field modality.

Importantly, the intention of the queried passage in the Discussion is not to claim that prior work has "poor resolution" compared to the present study. The section in full reads:

*"The most common approaches to X-ray nanotomography are scanning nanobeam (or nanoprobe) tomography and full-field nanotomography. Scanning X-ray nanoprobes offer high spatial resolution but are resolution-limited by X-ray focusing optics and are prohibitively slow (for in-situ measurements). Acquisition times scale poorly with sample volume and resolution due to the need for raster scanning via a resolution-limiting probe.[31,32] Full-field nanotomography enables faster volumetric imaging by capturing entire projections simultaneously, but is equally limited by optical constraints resulting in a trade-off between high-spatial resolution and large field-of-view. ~~As a result, **practical** resolutions are **typically** limited to several hundred nanometres to a few microns~~.[28-30,65,66]"*

This statement is intended as a general description of the practical trade-offs in existing scanning and full-field nanotomography implementations, not as a direct benchmark against our or other specific results. To avoid any possible misinterpretation, we have removed the final sentence of this paragraph.

5. Lines 63,96,284,285,377: The paper says near field ptychography but it really looks like far field ptychography with a zone plate. Can they explain how the near field measurements were made? It looks like they're measuring diffraction patterns rather than holograms. And there is no diffuser (that I can tell) which is required by near-field setups.

We thank the reviewer for raising this point and for prompting clarification of the experimental geometry.

(i) Near-field ptychography and the use of a diffuser.
A diffuser is not a general requirement for near-field ptychography. See X-ray near-field multi-slice ptychography for in-situ imaging, Sci. Rep. 15, 32150 (2025) and X-Ray Near-Field Ptychography for Optically Thick Specimens, Phys. Rev. Applied 3, 014005 (2015). In our experiment, the illumination was defined by a Fresnel zone plate with locally displaced zones, as stated in the Methods, to improve probe quality.

(ii) Near-field versus far-field regime.
The reviewer's question regarding the near-field designation is fully understandable given an **incorrect focus-to-sample distance stated in the original manuscript.** The correct distance was 6 mm (not 1.8 mm), resulting in a probe diameter of approximately 20 µm at the sample. Apologies for the oversight. For far-field ptychography, the maximum usable probe diameter is limited by the requirement that the speckles are sufficiently sampled at the detector for the given photon energy, sample–detector distance and detector pixel size. In our geometry (6.2 keV, detector at 5.229 m, 75 µm pixels), this condition would restrict the probe diameter to approximately 7 µm, we exceeded this limit and thus treated the data with a near-field propagator. Consistent with this, the effective pixel size in our reconstructions (~82 nm) is significantly larger than would be achievable under far-field ptychographic conditions (~15 nm). We have amended this part of the methods section:

*"**Tomogram Acquisition and Reconstruction**: NF-PXCT[33] experiments were carried out at 6.2 keV at the cSAXS beamline of the Swiss Light Source, Paul Scherrer Institut, Switzerland. The photon energy was selected using a double-crystal Si(111) monochromator. The horizontal aperture of slits located 22 m upstream of the sample was set to ~20 µm in width, to create a horizontal virtual source point that coherently illuminated a Fresnel zone-plate, 200 µm in diameter, with an outermost zone width of 60 nm. The Fresnel zone-plate was designed with locally displaced zones to improve imaging quality.[87] The focus to sample distance was 6 mm, producing a focused probe of ~20 µm diameter. Coherent diffraction patterns were acquired using an in-vacuum Eiger 1.5M area detector, with a 75 µm pixel size, placed 5.229 m downstream of the sample inside in an evacuated flight tube. Given the photon energy, sample to detector*

distance and detector pixel size, the far-field ptychography sampling condition would limit the maximum probe diameter to ~7 μm; the data were therefore reconstructed using a near-field propagator."

6. Lines 481-483: they should add more information about uncertainty. What is the local uncertainty in volumes that they are analyzing and assigning composition to? For example, the meta-stable phase.

The local uncertainty and phase separation are quantified in the manuscript via time- and temperature-resolved electron-density histograms of the relevant volumes, rather than by standard deviation or simple error bar referrals. These histograms show the full density distributions within the selected regions. The position of the (labeled) histograms peaks, their separation and overlap, allow a direct assessment of both the average phase densities, their spread and evolution over time and temperature. We believe this provides a more complete and accessible representation. The metastable phase and vaterite evolution figures in the main and SI showcase this. Overall electron-density uncertainty, and the spatial and temporal resolution estimates, are already provided in the Methods and SI.

In revisiting this point, we realized that the numerical segmentation thresholds used for phase assignment had not been stated. If the reviewer's question was directed at these thresholds, we apologize for the omission; they have now been added to the Methods, where we specify the electron-density ranges used for segmentation. The amended Methods sections read as follows:

"Within this isolated sub-volume, we then calculated the relative volumes of metastable polymorph, calcite, amorphous calcium carbonate (ACC), and pores at each time point using threshold-based segmentation. Voxels with an electron density < 0.15 n_e Å^{-3} were classified as pores. Voxels with densities between 0.15 and 0.41 n_e Å^{-3} were classified as dehydrated ACC. Voxels with densities between 0.41 and 0.75 n_e Å^{-3} correspond to metastable phase, and voxels with densities ≥ 0.75 n_e Å^{-3} were classified as calcite. Threshold selection was informed by the electron-density histograms shown in Figure 3c, which capture the local phase evolution with time and temperature."

"*Volume-Defect Mediated Calcite Growth and Vaterite Recrystallization:* The growth and transformation dynamics of vaterite (Figure S13) and defect-rich calcite (Figure 4), were quantified using the same volumetric analysis approach described for the metastable phase. Here, voxels with an electron density < 0.15 n_e Å^{-3} were classified as pores. Voxels with densities between 0.15 and 0.75 n_e Å^{-3} were classified as vaterite. Voxels with densities ≥ 0.75 n_e Å^{-3} were classified as calcite."

During this revision we also noticed that the x-axis in Figure S14b was inadvertently offset during figure assembly; this has been corrected in the updated version of the figure.

7. Lines 420-429: The analysis of the speed increase is incomplete. Certainly ptychography doesn't require 250000 times fewer photons than conventional scanning microscopy. So their estimate of the time for scanning probe is way too high since it shouldn't require 50ms per point. Any gain is mostly due to a slow scanning system which is just an engineering problem and not inherent to the imaging methods. I think the claim here should be softened.

We agree that this section required clarification and that our original back-of-the-envelope estimate was too simplistic. The demonstrated >100-fold gain in temporal resolution from the dynamic sparse reconstruction arises from a direct reduction in the required number of projections relative to the analytic sampling requirement (Crowther) and is independent of the specific projection acquisition modality. The initial comparison of out-of-focus X-ray ptychography to conventional scanning nanoprobe imaging, evaluated the possible projection speed advantage solely based on the number of required *scan points per projection*, ignoring photon efficiencies, which sig. overstates the practical gain.

In ptychography, spatial resolution is largely decoupled from scanning step size, enabling nm resolution from µm scan steps (144 points per 80×45 µm² projection here) rather than the ~300,000s points required by a 100 nm nanobeam raster scan of the same field of view. The initial estimate combined this ~2,500× scan point reduction with the 100× angular sampling gain from sparse reconstruction. Incorrectly assuming equal dwell times per scan point times. While ptychography is more photon-efficient per resolution element than classical scanning nanobeam imaging, current evaluations suggest a benefit of 10x fold and thus a good error in the initial statement. To avoid overstating the practical speed advantage, we have revised the section as follows:

"In comparison to analytic tomographic reconstruction methods,[31] which require approximately 1,250 projections to reconstruct a high-resolution tomogram of an 80 µm sample at 100 nm spatial resolution, the dynamic sparse reconstruction approach applied here achieves comparable image quality using only 10 projections per time point. … Further considering that X-ray ptychography can achieve up to an order-of-magnitude higher dose efficiency than conventional scanning nanoprobes at fixed spatial resolution and image quality and the ability to employ micrometre-scale scanning probes while retaining nm spatial resolution, this can reduce the number of scan positions required per resolution element by roughly one order of magnitude for the same field-of-view. When combined with the sparse angular sampling, this implies a substantial potential acquisition-speed advantage over conventional nanoprobe tomography for in-situ measurements of the same volume and resolution. The exact factor will depend on practical details such as source brightness, scan strategy and performance of utilized instrumentation."

8. Line 221-222: PXCT is not detecting defects, as fluorescence or diffraction would. PXCT has no direct sensitivity to the defects and their presence is rather guessed based on electron density which depends on many factors. This statement should be clarified.

We thank the reviewer for raising this point. PXCT does not measure defects directly in the same sense as diffraction or fluorescence. Rather, it provides quantitative 3D electron-density maps, and nanoscale defects are inferred from **local deviations in electron density**. This approach allows visualization of pores, segregated phases, and other volumetric microstructural features at the accessible scale.

To clarify this, we have revised the text to read:

"Imaging Thermal Coarsening and Defect Evolution in Single Crystals

In situ ptychographic nanotomography at elevated temperatures also enabled the visualization of nanoscale defects within crystals by the detection of local electron-density deviation, and their role in the Ostwald ripening of crystalline materials. "

9. line 55: change “statistical” to “statistically”

line 86: change “transformation” to “transformations”

Thank you for noticing, both were corrected.

Reviewer #3 (Remarks to the Author):

The authors present in their manuscript an in-situ monitoring of the crystallization process of the amorphous calcium carbonate (ACC) while heating up to 500 degrees C using near-field ptychography. Their measurements are followed by extensive data processing, reconstruction, and analysis, visualizing the different stages of crystallization. While the results are impressive – especially the combination of in-situ heating and the demonstrated spatial resolution – the manuscript lacks clarity in several aspects and requires clarification in a major revision.

Major concern: The focus of the manuscript remains unclear.

Is the crystallization process and its in-situ tracking over the temperature the key novelty? In this case, the manuscript should contain a clear motivation for focusing on the crystallization of amorphous calcium carbonate and why it is essential to visualize the different crystallization stages.

Is the focus much more on the imaging method? In this case, the focus must be sharpened to stress the impressive performance of the near-field ptychographic nanotomography in these in-situ experiments. I recommend not emphasizing the uniqueness in achieving the specifications, like the high temporal (minute-scale) and spatial (nanoscale) resolution, allowing for representative volume sizes. A closer look at the reported temporal resolution (5-25 minutes per tomogram), spatial resolution (130 nanometers), and the analyzed sample size (diameter 80 micrometersl) reveals that other nano-tomography techniques like for example, nearfield-holotomography or X-ray Zernike phase-contrast nanotomography, can achieve similar performance at the same or even larger sample sizes in similarly or even shorter times (see https://doi.org/10.1107/S1600577522012103 or https://doi.org/10.1364/BOE.396695). In-situ heating in combination with nanoscale imaging has already been demonstrated https://doi.org/10.1016/j.ceramint.2023.11.216. The in-situ study with near-field ptychographic nanotomography at this speed and resolution is the apparent novelty.

The advantages of using near-field ptychography to study the crystallization processes (e.g., accurate electron density mapping, resolving minimal changes in materials) should be emphasized more clearly. Also, more details are required on the choice of the amorphous calcium carbonate and why it is essential to visualize the different crystallization steps.

We thank the reviewer for this constructive comment regarding the focus of the manuscript.

The primary focus of the manuscript is the imaging methodology: an integrated platform for time- and temperature-resolved ptychographic nanotomography, combining quantitative 3D electron-density mapping, minute-scale temporal sampling, and representative sample volumes under controlled environmental conditions. The amorphous calcium carbonate (ACC) system is a secondary focus, chosen as a first investigation target not only to demonstrate the capabilities of this platform for capturing complex, multi-pathway crystallization processes but because of its scientific interest.

ACC is a widely studied mineral precursor in nature and industry and serves as a reference system for non-classical crystallization pathways involving multiple metastable phases and transformations across broad temperature and length scales. These characteristics make it particularly suitable for demonstrating a 4D imaging approach capable of resolving spatially heterogeneous and transient processes relevant to functional materials and manufacturing. **We have expanded the Introduction to make this rationale for ACC and its crystallization more explicit.**

We acknowledge that other nano-tomography techniques can reach comparable spatial resolution and acquisition times, and were already citing relevant examples in the manuscript. The reviewer mentioned examples have further been added. To clarify the positioning of our work, t**he Introduction and Discussion now explicitly situate our approach relative to existing methods, highlighting both advantages, current limitations and future development opportunities.**

Minor details:

1. Lines 115-116: The cited publication on the speed of analytical reconstruction methods is from 1970. The authors should cite a more recent work on tomographic reconstruction and compare the speed of their approach with the state-of-the-art tomographic reconstruction.

We thank the reviewer for this remark. The Crowther paper (Crowther R, A. The reconstruction of a three-dimensional structure from projections and its application to electron microscopy. *Proc. R. Soc. London A* 317, 319-340 (1970).) is cited here because it the "first" to provide an **analytical relation** between the number of projections, object size, and isotropic 3D resolution. In the context of our manuscript, it serves as a well-defined baseline for the angular sampling required by analytic reconstruction methods and thus enables a transparent comparison of our dynamic sparse reconstruction to any current or future tomographic reconstruction/ acquisition approach on the basis of a common analytical projection/ sampling requirement. It is not intended as a citation for state-of-the-art reconstruction methods, their dependencies and limitations. To avoid ambiguity, we explicitly refer to *analytic reconstruction methods* throughout, and we clarify that our comparison is made with respect to this analytical sampling baseline.

From the corresponding method section.

"Tomographic reconstruction was performed using a dynamic sparse reconstruction technique, as first described in Gao et. al.[37] *The technique allows dynamic tomography reconstruction with increased temporal resolution, which is comparable to the acquisition time of each sparsely sampled tomogram, i.e. each set of 10 projections, and without compromise in spatial resolution. …* ***In comparison to analytic tomographic reconstruction methods,***[31] *which require approximately 1,250 projections to reconstruct a high-resolution tomogram of an 80 µm sample at 100 nm spatial resolution, the dynamic sparse reconstruction approach applied here achieves comparable image quality using only 10 projections per time point."*

We added the following to the end of the paragraph to avoid possible future misunderstandings.

"A comprehensive benchmarking against the many existing iterative and compressed-sensing reconstruction methods is beyond the scope of this work; here we instead refer to the Crowther angular sampling criterion as a common analytical baseline for angular sampling, ensuring future comparativeness. "

2. Abstract and line 165: The term ‘Ostwald ripening’ needs an explanation. Please do not use special terms without introducing them.

Understood. We have added an explanation at the first mention of Ostwald ripening in the main text. The updated section reads as follows:

“At 250 and 500 °C, the calcite peak sharpens, and the histogram baseline gradually rises, which is consistent with the formation of calcite crystals and ongoing Ostwald ripening. The latter refers to a process in which larger crystals grow at the expense of smaller ones to reduce a system's surface energy (Note S2). Visually, as shown in Figure 2a, this manifests as the gradual disappearance of smaller crystals and the concurrent growth of larger ones within an increasingly porous sample volume. “

3. Lines 481ff: Electron Density Uncertainty is estimated by the standard deviation of the electron density of the surrounding air. This is only a good measure for the electron density uncertainty in a static process. In the dynamic process (heating and crystallization), the uncertainty of one voxel within the specimen is larger than that of the non-changing voxel with surrounding air. The voxels are averaging for 25 minutes over a changing sample region. This should increase the uncertainty.

We thank the reviewer for raising this important point and agree that uncertainty in dynamically evolving voxels is higher than in static regions such as surrounding air. In the present work, two distinct contributions to the electron-density uncertainty must therefore be distinguished.

The reported uncertainty of ~0.008 e^{-} $Å^{-3}$, estimated from the standard deviation of the electron density in the surrounding air, reflects the **baseline measurement uncertainty** associated with the ptychographic phase retrieval and reconstruction under static conditions, e.g. validity is restricted to sample areas changing significantly slower the tomogram acquisition time of 5 minutes. This value is appropriate for quantifying the instrumental and reconstruction-related noise floor. The second contribution arises from temporal averaging within voxels whose density changes during the acquisition window of a sparsely sampled tomogram. This contribution cannot be captured by a single universal number, because it depends on the (unobserved) intra-frame dynamics, which vary with material, transformation rate and temperature. A general problem of dynamic tomography.

To clarify this distinction, we have removed the simplified statement from the main text and revised the Methods section to explicitly state that the reported uncertainty derived from air represents a baseline value, and that dynamically changing voxels may exhibit higher effective uncertainty due to temporal averaging, bounded by the estimated temporal resolution of the experiment.

*"**Electron Density Uncertainty:** The measurement baseline voxel-level uncertainty in the reconstructed electron density was estimated by calculating the standard deviation (σ) of the electron density of air surrounding the sample across multiple time frames. This yields a sample-averaged uncertainty of approximately 0.008 e^{-} $Å^{-3}$, reflecting the measurement and reconstruction noise under static conditions and in non-changing sample regions. In dynamically evolving regions, additional uncertainty can arise from temporal averaging over the sub-tomogram acquisition window from on-going material transformation and relocation during acquisition. The magnitude of this contribution depends on the local transformation kinetics and experimental conditions and cannot be quantified by a single global value. Accordingly, the reported uncertainty from surrounding air should be interpreted as a baseline estimate, with higher effective uncertainty expected locally in rapidly changing voxels."*

4. Lines 521ff, Voxel-level Analysis: The authors should explain why a Principal Component Analysis allows for identifying the dominant crystallization patterns. This might not be clear to every reader.

We thank the reviewer for pointing this out. An explanation was indeed missing, in particular in the main text. We chose to amend the corresponding main text section as follows, being the readers first contact point, trying to remain brief and accessible to a general readership as much as possible. Happy to expand further if the editors or reviewers feel it is necessary.

"Finally, common transformation patterns and rare crystallization events were identified via voxel-level principal component analysis (PCA) and k-means clustering (Figure 2c & 13). PCA decomposes the multidimensional tomogram data into orthogonal components that capture the largest sources of variance, so that each component corresponds to a dominant, recurring pattern of electron-density change as a function of temperature and time. These analyses allow us to identify, isolate and quantify systematic changes that, in their sum, describe the multidimensional tomogram. The resulting patterns can then be assigned to specific transformation or crystallization events by considering, for example, their initial, intermediate and final electron densities, as well as their spatial distribution within the sample. Although most isolated patterns, Figure 3b&c, can be assigned to the ACC-to-calcite transition, the PCA further allowed us to capture rare events, including the presence and recrystallization of vaterite (see Note S3 and Figure S13), the formation and transformation of a hydrated metastable polymorph and the defect-driven ripening of calcite crystals, each discussed in detail below."
